# A ULK1–MTFR1L feedback loop links mitochondrial fission, mitophagy and apoptosis

**DOI:** 10.1242/jcs.264577

**Published:** 2026-05-11

**Authors:** Riccardo Babic, Leon Lucya, Christoph Reiter, Franziska Kriegenburg, Ramón Castellanos-Martínez, David M. Hollenstein, Florian Becker, Carola Hunte, Claudine Kraft

**Affiliations:** ^1^ Institute of Biochemistry and Molecular Biology, ZBMZ, Faculty of Medicine, University of Freiburg, 79104 Freiburg, Germany; ^2^ Faculty of Biology, University of Freiburg, 79104 Freiburg, Germany; ^3^ CIBSS - Centre for Integrative Biological Signalling Studies, University of Freiburg, 79104; ^4^ Mass Spectrometry Facility, Max Perutz Labs, Vienna Biocenter Campus (VBC), 1030 Vienna, Austria

**Keywords:** Autophagy, Mitophagy, MTFR1L, ULK1, ATG13

## Abstract

Mitophagy – the selective degradation of damaged mitochondria – preserves mitochondrial quality; yet, how mitochondrial fission is coordinated with autophagy initiation remains unclear. Here, we identified the mitochondrial outer membrane protein MTFR1L as a key component of mitophagy initiation hubs after using a synthetic FKBP–FRB system to tether ULK1 kinase to mitochondria independently of damage. We found that MTFR1L was enriched at ULK1 foci together with additional fission factors, and constitutive mitochondrial targeting of MTFR1L shifted mitochondrial morphology towards fragmentation. MTFR1L depletion decreased respiratory capacity, elevated apoptosis and impaired mitophagy flux. Upon mitophagy induction, MTFR1L was phosphorylated in a ULK1 kinase-dependent manner and reciprocally modulated ULK1 activity, establishing a feedback loop. Moreover, MTFR1L was required for proper ATG13 stability. These findings position MTFR1L as a critical link between mitochondrial fission and the autophagy machinery, coordinating mitophagy initiation and cell survival.

## Teaser

**Summary:** MTFR1L acts at mitochondrial mitophagy initiation sites, linking mitochondrial fission with autophagy by interacting with ULK1, regulating ATG13 stability, and forming a feedback loop that supports mitophagy and cell survival.

## INTRODUCTION

Mitochondria are dynamic organelles that undergo constant fusion, fission and selective removal through mitophagy – a selective form of autophagy essential for cellular homeostasis. This process involves the sequestration of damaged mitochondria into membranes, which mature into autophagosomes and deliver cargo to lysosomes (Onishi et al., 2021). Triggered by mitochondrial stress or damage, mitophagy proceeds through both ubiquitin-dependent and -independent pathways (Onishi et al., 2021; Ganley and Simonsen, 2022). In the ubiquitin-dependent route, PINK1 accumulates on damaged mitochondria and recruits Parkin from the cytosol (Narendra et al., 2008, 2010; Matsuda et al., 2010). Once phosphorylated by PINK1, Parkin ubiquitylates outer membrane proteins, forming a ubiquitin coat that recruits receptors such as NDP52 (also known as CALCOCO2) and OPTN (Lazarou et al., 2015). Such receptors link mitochondria to the autophagy machinery by interacting with the ULK1 complex component FIP200 (also known as RB1CC1), and TBK1 kinase further enhances this connection (Heo et al., 2015; Richter et al., 2016; Vargas et al., 2019). Ubiquitin-independent mitophagy occurs under conditions such as hypoxia or iron depletion via HIF1α-induced receptors such as BNIP3 and BNIP3L (also known as NIX) (Allen et al., 2013; Bellot et al., 2009; Tracy et al., 2007). These pathways also rely on ULK1 and FIP200, although details remain less defined (Munson et al., 2021; Yamashita et al., 2016).

Synthetic tethering of ULK1 to mitochondria is sufficient to trigger mitophagy independently of canonical stimuli and bypasses the requirement for receptors and TBK1 (Vargas et al., 2019; Eickhorst et al., 2024). ULK1 rapidly forms discrete foci across the mitochondrial network, serving as mitophagy initiation hubs that recruit downstream factors including FIP200, which connects the hub to the endoplasmic reticulum (ER) (Licheva et al., 2025). FIP200 remains essential in this context, indicating its key role in phagophore assembly site (PAS) maturation via ER–mitochondria contact sites (Eickhorst et al., 2024).

Although recruitment of receptors and the action of the ULK1 complex in mitophagy is increasingly understood, how PAS assembly is regulated and coordinated with mitochondrial fission remains unclear.

## RESULTS AND DISCUSSION

### Fission factors localize to mitophagy initiation hubs

**Fig. 1.**
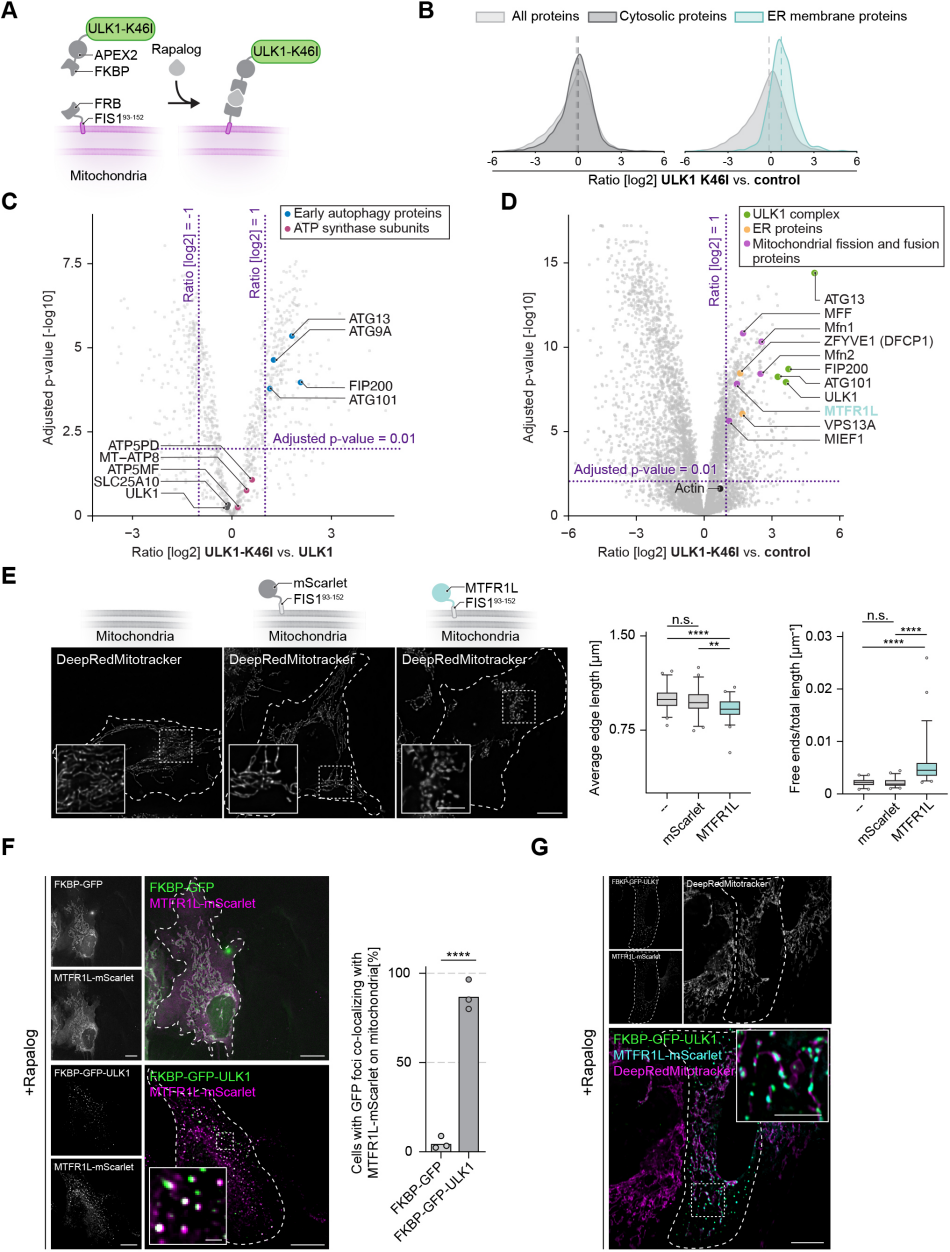
**Fission factors localize to mitophagy initiation hubs.** (A) Schematic of the synthetic tethering system. FRB is targeted to the outer mitochondrial membrane via fusion with the tail anchor domain of the mitochondrial membrane protein FIS1 (FRB–FIS1^93–152^). Co-expression of FKBP fusion proteins allows their rapalog-induced tethering to mitochondria. (B) After rapalog-induced tethering of stably expressed 2×FKBP–APEX2–ULK1-K46I or control 2×FKBP–APEX2 to FRB–FIS1^93–152^ (0.5 µM, for 2 h) in HEK293 cells, mass spectrometry analysis of APEX2-based proximity labeling was induced by the addition of biotin phenol and a short pulse of H_2_O_2_. Biotinylated proteins were enriched and analyzed using mass spectrometry. Kernel density estimate (KDE) plots displaying the distribution of protein abundance ratios for the ULK1-K46I mutant versus the control sample are shown. The plots compare the distribution of all quantified proteins with the subset of proteins annotated with the Gene Ontology (GO) term ‘cytosol’ (GO:0005829) or ‘endoplasmic reticulum membrane’ (GO: 0005789). The *y*-axis represents probability density. Tick marks and labels are omitted as absolute density values depend on smoothing bandwidth and are not directly interpretable. The plots are intended for qualitative comparison of distributional shape between groups. (C) Volcano plot of mass spectrometry data comparing the signal over control ratios of ULK1-specific proteins in the 2×FKBP–APEX2–ULK1-K46I versus 2×FKBP–APEX2–ULK1 WT condition. Dashed purple lines mark the threshold used to call proteins relatively enriched in the ULK1-K46I versus ULK1 condition (log2 ratio>1 and adjusted *P*-value<0.01). Early autophagy proteins meeting these criteria are highlighted in blue; selected mitochondrial proteins showing no significant change in this comparison are highlighted in pink. (D) Volcano plot showing enrichment of biotinylated proteins for 2×FKBP–APEX2–ULK1-K46I compared to 2×FKBP–APEX2. Dashed purple lines indicate cut-offs for ULK1-K46I-enriched proteins (log2 ratio>1 and adjusted *P*-value<0.01). A selection of enriched ULK1 complex proteins are highlighted in green, mitochondrial fission and fusion proteins are in purple, and endoplasmic reticulum (ER) proteins are in yellow. (E) Left: U2OS cells (cCE27 line) were transiently transfected with either FIS1^93–152^–mScarlet or FIS1^93–152^–MTFR1L. Mitochondria were stained using Mitotracker Deep Red. Representative images from one of three independent experiments are shown. Scale bars: 10 µm; 5 µm (inset). Right: mitochondrial network morphology was analyzed using Mitograph. Box plots represent average mitochondrial edge length (µm) and the ratio of free mitochondrial ends over total network length (µm^−1^). Boxes show the interquartile range, the whiskers indicate the 5th and 95th percentiles, and the median is marked with a line. A minimum of 50 cells were analyzed across replicates (*n*=3). Statistical analysis: one-way ANOVA followed by Sidak's multiple comparison test. (F) Left: U2OS cells (cCE308 line) stably expressing FRB–FIS1^93–152^ and transiently transfected with 2×FKBP–GFP–ULK1 or 2×FKBP–GFP, as well as MTFR1L–mScarlet, were cultured in nutrient-rich medium. Tethering was induced with 0.5 µM rapalog for 2 h before live-cell fluorescence microscopy. Representative images from one of three independent experiments are shown. Dashed lines indicate cell contours. Scale bars: 10 µm; 1 µm (inset). Right: quantification shows the percentage of cells with 2×FKBP–GFP–ULK1 foci colocalizing with MTFR1L-mScarlet on mitochondria from three independent experiments. Data are mean values. Circles show the mean value of each independent biological replicate (*n*=3). Bars show the mean of all replicates. A minimum of 80 cells per condition were analyzed across replicates. Statistical analysis: two-tailed unpaired *t*-test. (G) U2OS cells (cCE308 line) stably expressing FRB–FIS1^93–152^ and transiently co-transfected with 2×FKBP–GFP–ULK1 and MTFR1L–mScarlet were treated with 0.5 µM rapalog for 2 h. Mitochondria were stained with MitoTracker Deep Red prior to imaging. Live-cell fluorescence microscopy shows the mitochondrial network (MitoTracker Deep Red), MTFR1L–mScarlet distribution and FKBP-GFP-ULK1 foci. A representative image from three independent experiments is shown. Scale bars: 10 µm; 5 µm (inset). n.s., not significant; **P<0.01; ****P<0.0001.

To study the local environment of initiation hubs during mitophagy, we previously used APEX2-based proximity labeling in combination with a rapalog-inducible FKBP–FRB dimerization system in U2OS cells. FRB was fused to the tail anchor domain of the mitochondrial membrane protein FIS1 (FRB–FIS1^93–152^). Co-expression of an FKBP–GFP–ULK1 fusion enabled rapalog-dependent tethering to mitochondrial FRB–FIS1^93–152^ and thereby triggers mitophagy without causing mitochondrial damage (Fig. 1A) (Vargas et al., 2019; Banaszynski et al., 2006; Torggler et al., 2016; Eickhorst et al., 2024; Licheva et al., 2025). Using this approach, we previously demonstrated that tethering FKBP–APEX2–ULK1 to mitochondria leads to the formation of distinct mitophagy initiation hubs that recruit downstream autophagy factors. Among these factors is FIP200, which is required for the efficient connection to the ER, where PAS maturation and ultimately phagophore nucleation take place (Licheva et al., 2025). Moreover, FIP200 promotes downstream autophagy events, supporting efficient autophagosome biogenesis (Eickhorst et al., 2024).

We next asked whether ULK1 kinase activity is required for the establishment of the ER connection and repeated the proximity labeling with the kinase-dead ULK1 variant FKBP–APEX2–ULK1-K46I, followed by mass spectrometric (MS) analysis of biotinylated proteins. Similar to the active version, FKBP–APEX2–ULK1-K46I enriched biotinylated ER proteins compared to FKBP–APEX2, but not cytosolic proteins (Fig. 1B) (Licheva et al., 2025), indicating that ULK1 kinase activity is not necessary for ER engagement but that it acts downstream of establishing this connection. Notably, comparing FKBP–APEX2–ULK1-WT to FKBP–APEX2–ULK1-K46I revealed an increase in proximity labeling of PAS-associated factors such as ULK1 complex components and ATG9A, beyond the enrichment already observed for FKBP–APEX2–ULK1-WT (Fig. 1C) (Licheva et al., 2025). In contrast, the labeling of general mitochondrial proteins such as ATP synthase subunits (ATP5PD, ATP5MF and MT-ATP8) or the mitochondrial dicarboxylate carrier SLC25A10 remained unchanged (Fig. 1C). This indicates that ULK1-K46I effectively stalls mitophagy at an early stage after the ER connection has been established, promoting the accumulation of factors involved in PAS formation and mitophagy initiation, without broadly affecting general mitochondrial protein distribution.

Synthetic ULK1 tethering to mitochondria activates mitophagy independently of damage signals, implying that fission must occur in parallel or downstream of ULK1 recruitment (Eickhorst et al., 2024). Among the biotinylated proteins enriched with FKBP–APEX2–ULK1-K46I, we detected several fusion factors, including Mfn1 and Mfn2, as well as fission factors such as MFF and MIEF1. We also identified MTFR1L, a less-characterized outer membrane protein implicated in mitochondrial morphology dynamics (Fig. 1D) (Tilokani et al., 2022). In the absence of MTFR1L, mitochondria were found to exhibit hyperfusion, forming a highly interconnected network. Knockdown of MTFR1L has been reported to neither alter the recruitment of the key fission protein DRP1 (also known as DNM1L) to mitochondria, nor change the cellular abundance of DRP1 or its receptors MFF and MiD49 or MiD51. Although MTFR1L depletion also does not affect the levels of the outer membrane fusion proteins Mfn1 and Mfn2, it was observed to increase the levels of the inner mitochondrial fusion factor OPA1 and was suggested to function as a negative regulator of fusion events (Tilokani et al., 2022).

To test whether MTFR1L is also able to promote fission, we fused MTFR1L to the outer mitochondrial membrane anchor of FIS1 (FIS1^93–152^–MTFR1L). As a control, we expressed FIS1^93–152^–mScarlet. Quantification of mitochondrial network morphology using MitoGraph analysis (Viana et al., 2015) revealed a significant reduction in average mitochondrial edge length and an increase in the ratio of free mitochondrial ends to total network length in cells expressing FIS1^93–152^–MTFR1L compared to those in control cells (Fig. 1E; Fig. S1). This indicates a shift towards a more fragmented mitochondrial network, supporting a role of MTFR1L in directly shaping mitochondrial dynamics.

We next asked whether MTFR1L associates with the autophagy machinery and observed that MTFR1L–mScarlet colocalized with FKBP–GFP–ULK1 foci following synthetic tethering (Fig. 1F), suggesting that MTFR1L is part of ULK1 hubs on mitochondria. To further characterize this localization, we performed live microscopy visualizing the mitochondria network. The MitoTracker Deep Red dye revealed an intact, spread mitochondrial network, whereas MTFR1L–mScarlet was enriched in discrete foci that colocalized with FKBP–GFP–ULK1 upon rapalog treatment (Fig. 1G). This demonstrates that MTFR1L–mScarlet marks specific ULK1-positive initiation hubs rather than the mitochondrial network as a whole, and that its punctate appearance does not reflect mitochondrial fragmentation.

### MTFR1L is required for mitochondrial respiration and cell fitness

**Fig. 2.**
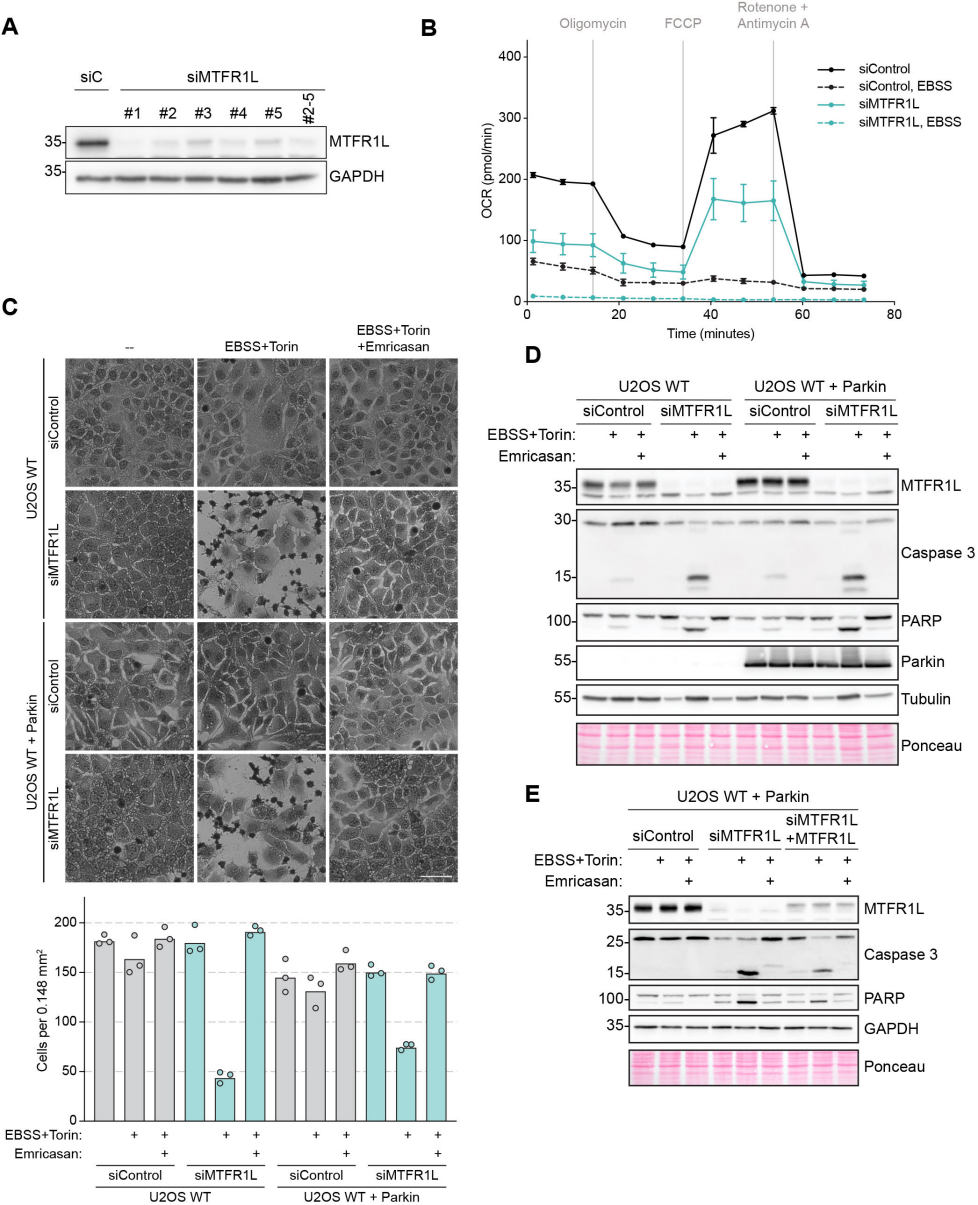
**MTFR1L is required for mitochondrial respiration and cell fitness.** (A) U2OS cells (cCE308 line) stably expressing FRB–FIS1^93–152^ were treated for 72 h with control siRNA, five individual siRNAs targeting MTFR1L (#1–5) or a pooled combination of siRNAs #2–5. Cells were lysed and depletion of MTFR1L was assessed by western blotting. (B) U2OS cells (cCE308 line) stably expressing FRB–FIS1^93–152^ were treated for 72 h with control or MTFR1L siRNA #1. Cells were cultured in nutrient-rich medium or transferred to starvation medium (EBSS) for 4 h before harvesting. The cells were then analyzed for mitochondrial oxygen consumption rates (OCRs), using the Agilent Seahorse XF Cell Mito Stress Test protocols. Oligomycin, FCCP and rotenone/antimycin A were added according to the manufacturer's instructions. Data are shown as mean±s.d. of three independent biological replicates. (C) Top: U2OS cells, with (cPS66 line) and without (cCE377 line) stable Parkin expression, were treated with control siRNA or MTFR1L siRNA #1. Autophagy was induced by culturing the cells in starvation medium (EBSS) supplemented with Torin 1. Cell viability was assessed by Crystal Violet staining and visualized via microscopy. Where indicated, cells were treated with 1 µM Emricasan (pan-caspase inhibitor) for 4 h. Representative images from one of three independent experiments are shown. Scale bar: 50 µm. Bottom: the graph shows the mean cell number per field of view (0.148 mm^2^, 20× objective) from three independent biological replicates. Each circle represents the mean value of one biological replicate. (D) U2OS cells, with (cPS66 line) and without (cCE377 line) stable Parkin expression, were treated with control siRNA or MTFR1L siRNA #1. The cells were cultured in nutrient-rich medium or transferred to starvation medium (EBSS) supplemented with Torin 1 for 4 h. Where indicated, cells were treated with the pan-caspase inhibitor Emricasan for 4 h. Cells were lysed and induction of apoptosis was assessed by western blotting following caspase-3 and PARP cleavage. Blots represent three independent experiments. (E) U2OS cells with stable Parkin expression (cPS66 line) were treated with control siRNA or MTFR1L siRNA #1 for 72 h. Where indicated, untagged MTFR1L was re-expressed by transient transfection. Cells were cultured in starvation medium (EBSS) supplemented with Torin 1 for 4 h. Where indicated, cells were treated with 1 µM Emricasan (pan-caspase inhibitor) for 4 h. Cells were lysed and induction of apoptosis was assessed by western blotting following caspase-3 and PARP cleavage. Blots represent three independent experiments.

As MTFR1L influences mitochondrial morphology (Fig. 1E), we further investigated the role of MTFR1L in mitochondrial function and depleted the MTFR1L protein using siRNAs targeting different sequences within the *MTFR1L* mRNA (Fig. 2A). All siRNAs successfully decreased MTFR1L protein levels. As MTFR1L siRNA #1 was the siRNA previously published (Tilokani et al., 2022), we performed subsequent analyses using this siRNA. We next tested cellular respiration using a Seahorse extracellular flux analyzer. MTFR1L depletion revealed a marked reduction in oxygen consumption, suggesting severe mitochondrial dysfunction (Fig. 2B). Consistent with this finding, *MTFR1L* silencing resulted in cell death (Fig. 2C), accompanied by increased apoptosis, as shown by elevated PARP (PARP1) and caspase-3 cleavage (Fig. 2D). Additional treatment with the antiapoptotic component Emricasan rescued caspase-dependent cell death in MTFR1L-depleted cells. These effects were observed in both Parkin-expressing and Parkin-deficient cells.

The increase in cleaved caspase-3 and PARP levels upon MTFR1L depletion is consistent with activation of the intrinsic, mitochondria-driven apoptotic pathway. Given the marked reduction in respiratory capacity observed in MTFR1L-depleted cells (Fig. 2B), we speculated that impaired mitochondrial bioenergetics lowers the apoptotic threshold. Re-expression of MTFR1L substantially reduced the levels of cleaved caspase-3 and cleaved PARP compared to their levels upon siMTFR1L treatment (Fig. 2E), demonstrating that the apoptotic phenotype is specifically caused by MTFR1L depletion and not by off-target siRNA effects.

Notably, our siRNA-mediated knockdown of MTFR1L resulted in effects opposite to those reported in previous knockout studies concerning caspase activity (Tilokani et al., 2022). We observed pronounced cellular toxicity upon prolonged depletion and were only able to obtain a very limited number of knockout clones in our hands, underscoring potential challenges with complete loss of MTFR1L and/or adaptation. Therefore, we focused on siRNA-based approaches, which allowed more consistent and interpretable analyses of MTFR1L function under less deleterious conditions.

Altogether, these findings indicate that MTFR1L is critical for maintaining mitochondrial function and cell viability.

### MTFR1L is required for mitophagy

**Fig. 3.**
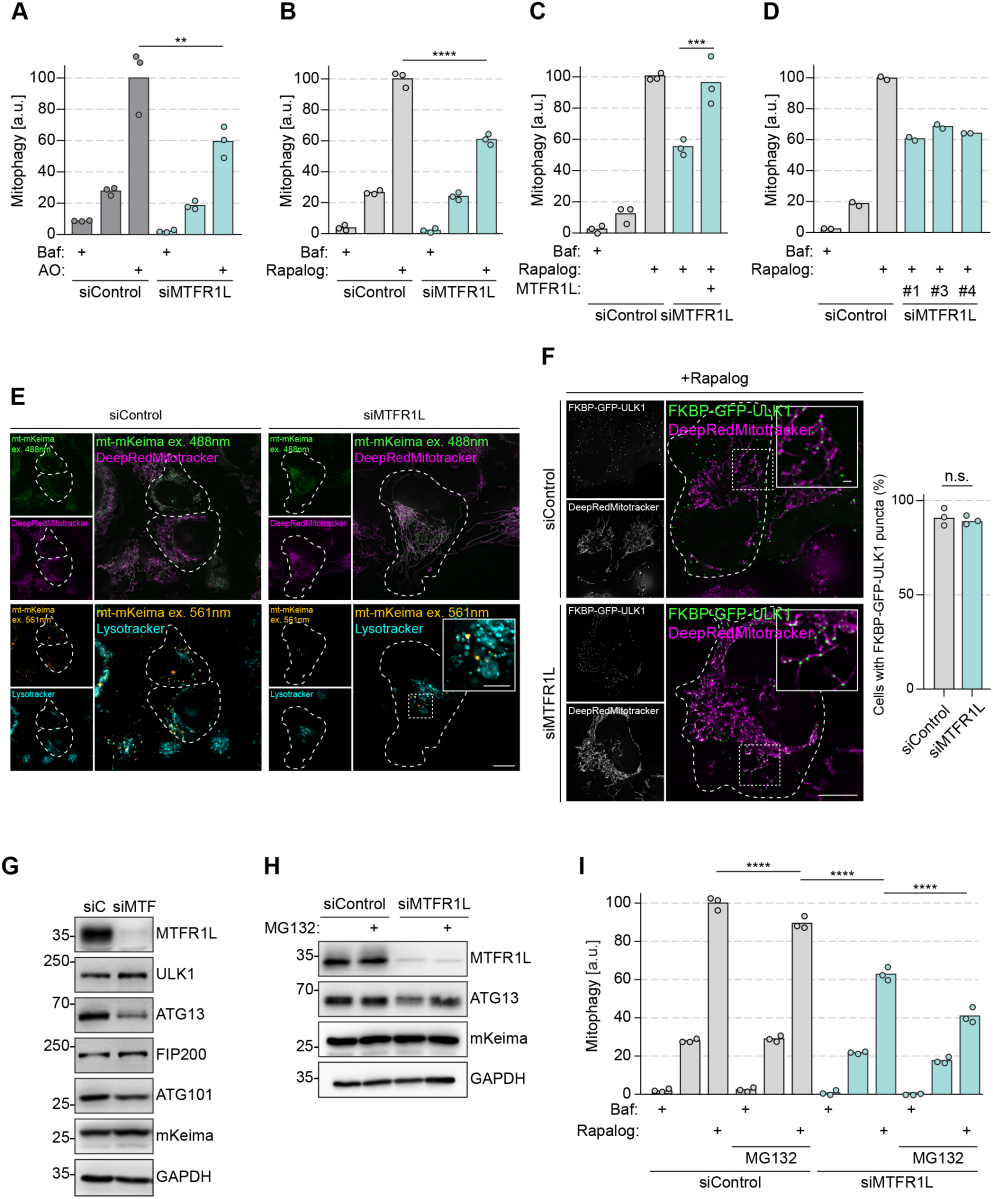
**MTFR1L is required for mitophagy.** (A) U2OS cells (cPS66 line) stably expressing FRB–FIS1^93–152^, mt-mKeima and Parkin were treated with control siRNA or MTFR1L siRNA #1 for 72 h. Where indicated, cells were treated with 200 nM bafilomycin A1 (Baf) for 4 h. Parkin-dependent mitophagy was induced with 4 µM of antimycin A and 4 µM of oligomycin (AO) for 4 h. Lysosomal mt-mKeima (561 nm) to cytosolic mt-mKeima (488 nm) ratios were analyzed by flow cytometry from three independent biological replicates. Bars show the mean of three independent biological replicates. Each circle represents the mean value of one biological replicate. Data are shown as normalized ratios of lysosomal to cytosolic mt-mKeima. Statistical analysis: one-way ANOVA followed by Sidak's multiple comparison test. (B,C) U2OS cells (cCE175 line) stably expressing FRB–FIS1^93–152^ and mt-mKeima were transiently transfected with 2×FKBP–GFP–ULK1. The cells were treated with control siRNA or MTFR1L siRNA #1 for 72 h. Mitophagy was induced by tethering FKBP–GFP–ULK1 to FRB–FIS^93–152^ with 0.5 µM rapalog for 24 h. Where indicated, the cells were treated with bafilomycin A1 as in A. Mitophagy flux was assessed and analyzed as in A. In C, MTFR1L-depleted cells were transfected with untagged MTFR1L to assess rescue. (D) U2OS cells (cCE175 line) stably expressing FRB–FIS1^93–152^ and mt-mKeima were transiently transfected with 2×FKBP–GFP–ULK1. To assess whether the mitophagy defect was specific to the previously published siRNA, individual siRNAs targeting MTFR1L for 72 h (#1, #3 and #4) were tested. Mitophagy was induced by tethering FKBP–GFP–ULK1 to mitochondrial FRB–FIS1^93–152^ with 0.5 µM rapalog for 24 h. Where indicated, cells were treated with bafilomycin A1 as in A. Mitophagy flux was assessed and analyzed as in A. (E) U2OS cells (cCE308 line) stably expressing FRB–FIS1^93–152^ were treated with control siRNA or MTFR1L siRNA #1 for 72 h. Cells were imaged by live-cell fluorescence microscopy under nutrient-rich conditions. Neutral-pH mt-mKeima signal (488 nm excitation) was co-stained with MitoTracker Deep Red to confirm mitochondrial localization. Acidic mt-mKeima puncta (561 nm excitation) were co-stained with LysoTracker to confirm lysosomal localization. Images represent three independent experiments. Scale bars: 10 µm; 5 µm (inset). (F) Left: U2OS cells (cCE308 line) stably expressing FRB–FIS1^93–152^ were transiently transfected with 2×FKBP–GFP–ULK1 and treated with control siRNA or MTFR1L siRNA #1 for 72 h. Cells were stained with MitoTracker Deep Red. Mitophagy was induced with 1 h treatment of 0.5 µM rapalog. Scale bars: 10 µm; 1 µm (inset). Right: quantification shows the percentage of cells with GFP foci colocalizing with mitochondria. Data are mean values. Circles show the mean value of each independent biological replicate (*n*=3). Bars show the means of all replicates. Statistical analysis: two-tailed unpaired *t*-test. (G) U2OS cells (cCE377 line) stably expressing FRB–FIS1^93–152^, mt-mKeima and 2×FKBP–GFP–ULK1 were treated with control siRNA or MTFR1L siRNA #1 for 72 h. Cells were lysed and the protein levels of ULK1 complex members were assessed by western blotting. Quantification is shown in Fig. S1B. Blots represent three independent experiments. (H) U2OS cells (cCE377 line) stably expressing FRB–FIS1^93–152^, mt-mKeima and 2×FKBP–GFP–ULK1 were treated with control siRNA or MTFR1L siRNA #1 for 72 h. Where indicated, cells were treated with 10 µM MG132 (proteasome inhibitor) for 4 h. Cells were lysed and ATG13 protein levels were followed by western blotting. Quantification is shown in Fig. S1C. Blots represent three independent experiments. (I) U2OS cells (cCE377 line) stably expressing FRB–FIS1^93–152^, mt-mKeima and 2×FKBP–GFP–ULK1 were treated with control siRNA or MTFR1L siRNA #1 for 72 h. To induce the expression of 2×FKBP–GFP–ULK1, cells were treated with doxycycline (1 µg/ml, 24 h) before flow cytometry data acquisition. Mitophagy was induced by tethering FKBP–GFP-ULK1 to FRB–FIS1^93–152^ with 0.5 µM rapalog for 24 h. Where indicated, the cells were treated with bafilomycin A1 as in A or with 10 µM MG132 for 4 h. Flow cytometry data were analyzed and quantified as described in A. a.u., arbitrary units. n.s., not significant; **P<0.01; ***P<0.001; ****P<0.0001.

The reduced oxygen consumption rate and increased caspase-3 cleavage indicate the accumulation of damaged mitochondria after silencing of *MTFR1L*. We therefore analyzed whether MTFR1L is required for mitophagy, as a functional link between MTFR1L and mitophagy has not yet been established. We examined whether MTFR1L influences the mitophagy flux using mitochondrial-targeted mKeima (mt-mKeima), a fluorophore with dual excitation properties that distinguishes between neutral and acidic pH environments. When targeted to mitochondria, mKeima enables detection of mitolysosome formation by flow cytometry and thus serves as a reliable readout of mitophagy flux. As a control, we used bafilomycin A1, which inhibits mitophagosome–lysosome fusion and prevents acidification of mitolysosomes (Katayama et al., 2011). In control cells, basal mitophagy was detectable under nutrient-rich conditions and increased upon induction with antimycin A and oligomycin (AO) as expected. In contrast, MTFR1L depletion reduced the mitophagy flux both under basal conditions and following AO treatment (Fig. 3A). A similar reduction was observed in MTFR1L siRNA-treated cells when mitophagy was triggered using the synthetic induction system tethering FKBP–GFP–ULK1 to mitochondrial FRB–FIS1^93–152^ by rapalog addition, compared to that in the mock control (Fig. 3B). Re-expression of MTFR1L rescued the mitophagy defect (Fig. 3C). A similar defect was observed when using other siRNAs for MTFR1L depletion (Fig. 3D). To ensure that the change in mt-mKeima excitation was not a secondary effect of MTFR1L depletion, we verified by fluorescence microscopy that the neutral-pH mt-mKeima signal overlapped with MitoTracker Deep Red signals under basal conditions in both siControl and siMTFR1L cells and confirmed mitochondrial delivery (Fig. 3E). In contrast, the acidic mt-mKeima population solely colocalized with LysoTracker-positive structures, validating lysosomal delivery. This demonstrates that MTFR1L is essential for maintaining efficient mitophagy flux and that it likely acts downstream of ULK1 recruitment to mitochondria. Furthermore, because mitochondrial quality control limits the accumulation of dysfunctional mitochondria, the mitophagy defect observed upon MTFR1L depletion might further increase apoptotic susceptibility (Fig. 2C,D) due to an increased amount of damaged mitochondria.

### MTFR1L interacts with the ULK1 complex

As MTFR1L was detected at mitophagy initiation hubs in close proximity to ULK1 (Fig. 1D), we asked whether it affects hub formation and ULK1 complex assembly. Depletion of MTFR1L by siRNA did not affect hub formation by ULK1 after rapalog treatment (Fig. 3F), indicating that MTFR1L functions downstream of ULK1-induced hub formation. Surprisingly, although the steady-state levels of ULK1, FIP200 and ATG101 levels remained unchanged upon MTFR1L knockdown, ATG13 abundance decreased (Fig. 3G; Fig. S1B). This suggests that ATG13 is stabilized by MTFR1L. ATG13 levels were restored by proteasome inhibition with MG132 (Fig. 3H; Fig. S1C), which, however, did not rescue the mitophagy flux but rather exacerbated the defect (Fig. 3I) as previously described (Tanaka et al., 2010), indicating that the flux defect is independent of ATG13 loss.

### ULK1 phosphorylates MTFR1L upon mitophagy induction

**Fig. 4.**
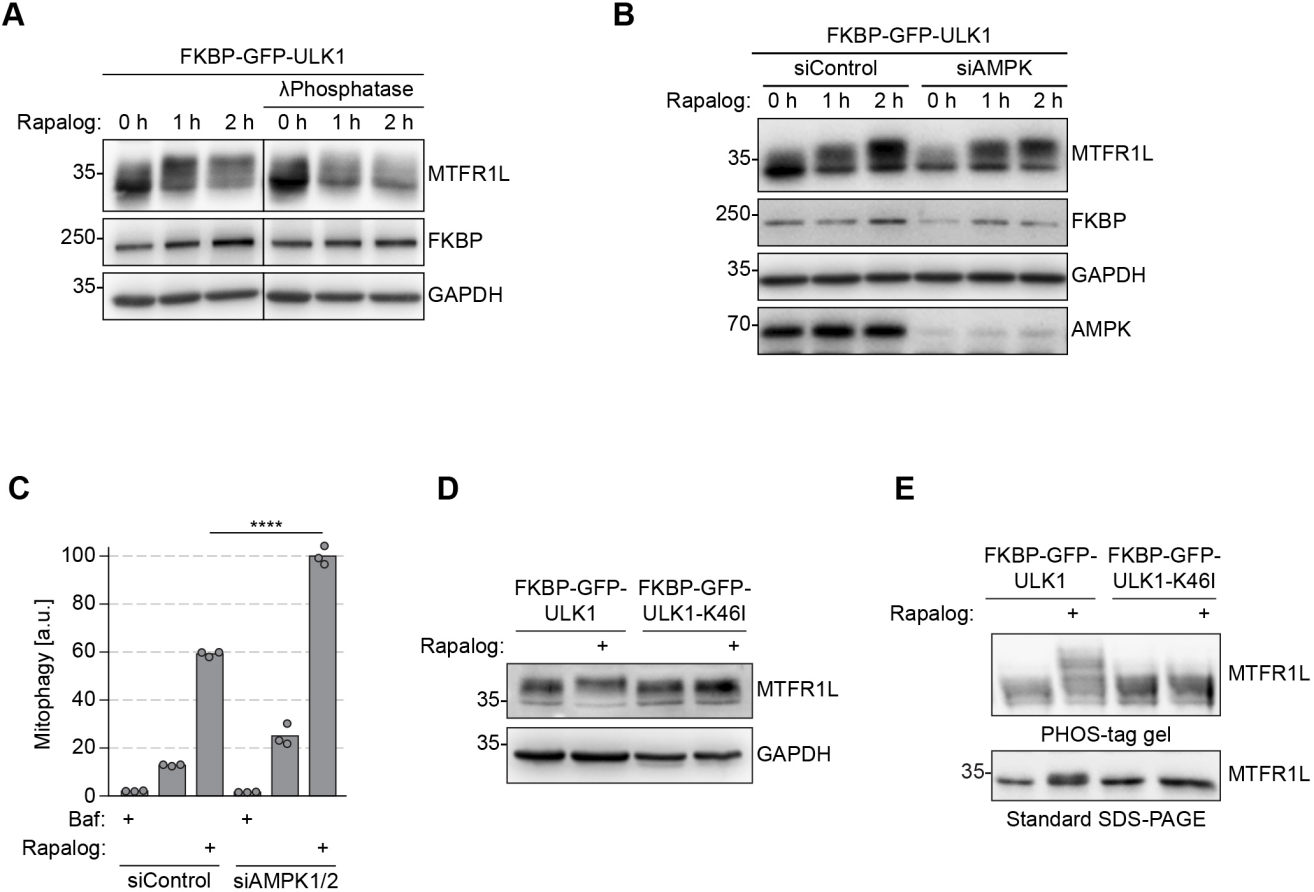
**ULK1 phosphorylates MTFR1L upon mitophagy induction.** (A) U2OS cells (cCE377 line) stably expressing FRB–FIS1^93–152^, mt-mKeima and 2×FKBP–GFP–ULK1 were treated with doxycycline (1 µg/ml, 24 h) to induce expression of 2×FKBP–GFP–ULK1. Cells were then treated with 0.5 µM rapalog for 1 or 2 h before harvesting. Cells were lysed and, where indicated, lysates were treated with λ phosphatase. MTFR1L was detected by western blotting, with FKBP and GAPDH as controls. (B) U2OS cells (cCE377 line) stably expressing FRB–FIS1^93–152^, mt-mKeima and 2×FKBP–GFP–ULK1 were treated with control or AMPK1/2 siRNA for 72 h. To induce expression of 2×FKBP–GFP–ULK1, cells were treated with doxycycline (1 µg/ml, 24 h). Cells were then treated with 0.5 µM rapalog for 1 or 2 h before harvesting. Cells were lysed and MTFR1L, AMPK, FKBP and GAPDH levels were analyzed by western blotting. (C) Cells as in B were grown and treated as in B. Where indicated, cells were treated with 0.5 µM rapalog or 200 nM bafilomycin A1 (Baf) for 4 h. Flow cytometry data were analyzed and quantified as described in Fig. 3A. a.u., arbitrary units. ****P<0.0001. (D) U2OS cells (cCE377 line) stably expressing FRB–FIS1^93–152^, mt-mKeima and 2×FKBP–GFP–ULK1, and U2OS cells (cCE403 line) stably expressing FRB–FIS1^93–152^, mt-mKeima and 2×FKBP–GFP–ULK1-K46I were treated with doxycycline (1 µg/ml, 24 h) to induce expression of 2×FKBP–GFP–ULK1 or 2×FKBP–GFP–ULK1-K46I. Cells were treated with 0.5 µM rapalog for 1 h before harvesting. Cell lysates were analyzed by SDS-PAGE, followed by western blotting. (E) Cell lysates prepared as in D were analyzed by Phos-tag gel electrophoresis or standard SDS-PAGE, followed by western blotting. Blots represent two independent experiments.

When analyzing MTFR1L by western blotting, we observed a mobility shift of MTFR1L after mitophagy initiation by ULK1 tethering. This shift was reversed by phosphatase treatment, indicating phosphorylation of MTFR1L (Fig. 4A). Previous phosphoproteomic studies identified MTFR1L as a potential AMPK substrate and proposed that AMPK-mediated phosphorylation is required for its proper function (Chen et al., 2019; Schaffer et al., 2015; Tilokani et al., 2022). In our synthetic mitophagy induction system, upstream signaling events are bypassed and AMPK signaling is not expected to play a major role. Nevertheless, we tested whether AMPK was responsible for the observed MTFR1L mobility shift. AMPK depletion had no effect on the shift, suggesting that MTFR1L is regulated by other kinases during mitophagy (Fig. 4B). Additionally, mitophagy flux was slightly enhanced rather than reduced upon AMPK depletion (Fig. 4C), consistent with previous reports describing AMPK as a negative regulator of ULK1 in upstream signaling events (Park et al., 2023). Taken together, these findings argue against AMPK being the relevant kinase regulating MTFR1L in this context.

As MTFR1L was found in close proximity to ULK1 (Fig. 1D), we asked whether ULK1 could be responsible for the observed phosphorylation shift. Indeed, when kinase-dead ULK1-K46I was tethered in place of ULK1-WT, the shift was no longer detectable (Fig. 4D). This was additionally confirmed using Phos-tag gels, in which MTFR1L shifted up in the presence of ULK1-WT but not ULK1-K46I (Fig. 4E). This suggests that ULK1, either directly or indirectly, mediates MTFR1L phosphorylation upon mitophagy induction.

**Fig. 5.**
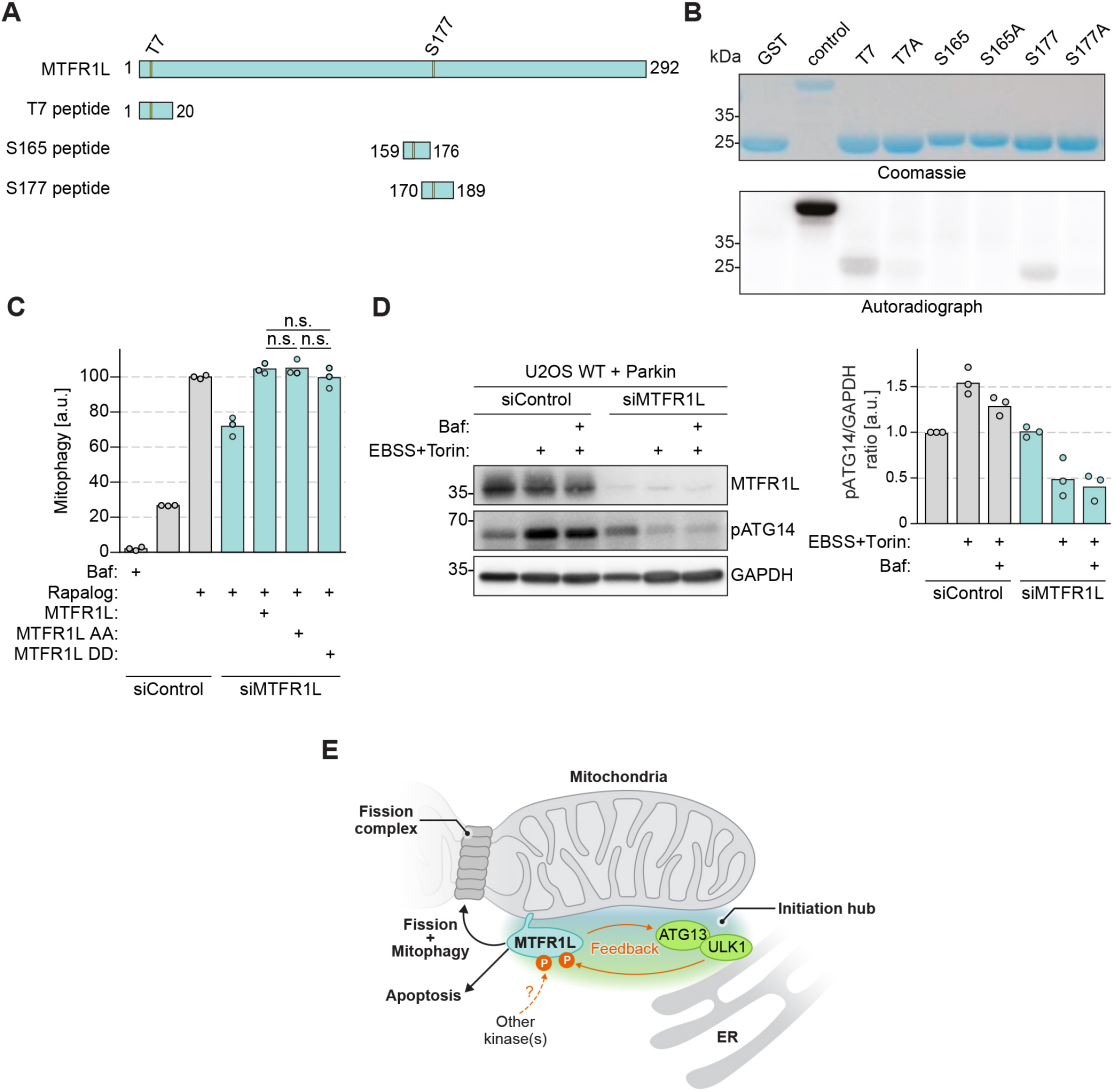
**ULK1 phosphorylates MTFR1L at threonine 7 and serine 177.** (A) Schematic of MTFR1L highlighting two residues matching the ULK1 consensus motif: threonine 7 (T7) and serine 177 (S177). Constructs used in the *in vitro* kinase assay (B) are illustrated. (B) GST fusion proteins were subjected to *in vitro* phosphorylation with recombinant ULK1: GST (negative control), GST–ATG19 C-terminus (positive control), GST–MTFR1L peptide fragments or their corresponding alanine mutants depicted in A [1–20 amino acids (aa), T7 or T7A; 159–171 aa, S165 or S165A; 170–189 aa, S177 or S177A] were used. One representative experiment out of two is shown. (C) U2OS cells (cCE175 line) stably expressing FRB–FIS1^93–152^ and mt-mKeima were treated with control siRNA or MTFR1L siRNA #1 for 72 h and were transiently transfected with 2×FKBP–GFP–ULK1. Where indicated, MTFR1L-WT, MTFR1L-T7A/S177A (AA) or MTFR1L-T7D/S177D (DD) were re-expressed by transient transfection. Flow cytometry data were analyzed and quantified as described in Fig. 3A. a.u., arbitrary units. n.s., not significant. (D) U2OS cells (cPS66 line) stably expressing FRB–FIS1^93–152^, mt-mKeima and Parkin were treated with control siRNA or MTFR1L siRNA #1 for 72 h. Where indicated, cells were transferred to starvation medium (EBSS) supplemented with Torin 1 for 4 h, and where indicated, treated with Bafilomycin A1 as previously described. Cells were lysed and analyzed by western blotting. Phosphorylated (p)ATG14/GAPDH ratio was quantified using signal intensity from three independent biological replicates. (E) Proposed model of MTFR1L function at mitophagy initiation hubs. MTFR1L localizes to the mitochondrial outer membrane where it contributes to the regulation of mitochondrial fission and mitophagy. Upon mitophagy induction, ULK1 phosphorylates MTFR1L. MTFR1L, in turn, supports ULK1 kinase activity and stabilizes ATG13, a core ULK1 complex component. This mutual regulatory interaction promotes mitophagy progression. Loss of MTFR1L disrupts this feedback loop, leading to impaired mitophagy, mitochondrial dysfunction and activation of apoptotic pathways. MTFR1L plays a role in mitochondrial dynamics, autophagy progression and cellular homeostasis.

MTFR1L contains two residues that match the ULK1 consensus motif: threonine 7 (T7) and serine 177 (S177) (Fig. 5A) (Egan et al., 2015; Mercer et al., 2021; Papinski et al., 2014). To test whether ULK1 directly phosphorylates MTFR1L, we generated GST fusion proteins containing peptides encompassing these sites and performed *in vitro* phosphorylation assays using recombinant ULK1. We detected phosphorylation for both sites, which was lost when these sites were mutated to alanine. S177 is present in a serine/threonine-rich patch within MTFR1L. To confirm the specificity of the kinase assay, we also probed serine 165 (S165) present in the same patch. S165 could not be phosphorylated by ULK1 *in vitro*, highlighting the validity of the assay (Fig. 5B). These results demonstrate that MTFR1L can be directly phosphorylated by ULK1. To assess the functional importance of these phosphorylation sites, we re-expressed wild-type MTFR1L, the phosphorylation-deficient double alanine mutant MTFR1L-T7A/S177A and the phosphomimetic double aspartate mutant MTFR1L-T7D/S177D in siMTFR1L-treated cells and monitored the mitophagy flux (Fig. 5C). All three constructs rescued the mitophagy defect to similar levels, suggesting that phosphorylation at T7 and S177 plays a modulatory rather than an essential role in MTFR1L function. Given that MTFR1L contains 52 serine and threonine residues in total, and has previously been identified as an AMPK substrate, it is conceivable that further phosphorylation events by ULK1 or additional kinases contribute to its regulation, potentially integrating multiple upstream signals at mitophagy initiation hubs. Future studies will be required to systematically map MTFR1L phosphorylation sites and clarify how ULK1 and potentially other kinases regulate its activity.

As MTFR1L associates with the ULK1 complex and is phosphorylated by ULK1, we asked whether MTFR1L might, in turn, influence ULK1 activity. To address this, we examined ATG14, a well-characterized direct target of ULK1 and a reliable readout of its kinase activity (Park et al., 2016; Wold et al., 2016). As expected, ATG14 phosphorylation increased in U2OS cells upon starvation. However, this phosphorylation was markedly reduced when MTFR1L was depleted, in line with MTFR1L modulating ULK1 kinase activity (Fig. 5D). Taken together, these findings support a mechanism in which MTFR1L and ULK1 mutually regulate each other to balance mitophagy and apoptosis (Fig. 5E).

Overall, our findings identify MTFR1L as a novel regulator of mitophagy that integrates mitochondrial fission and autophagy. MTFR1L localizes to mitophagy initiation sites with the ULK1 complex. Consistent with this, a concurrent study independently identified MTFR1L to be required for PINK1/Parkin-dependent mitophagy in the aging heart (Shi et al., 2026). MTFR1L influences ULK1 kinase activity under autophagy-inducing conditions and is itself a ULK1 substrate, suggesting reciprocal regulation (Fig. 5E). Loss of MTFR1L destabilizes ATG13, impairs mitophagy and enhances apoptotic signaling. Notably, ATG13 but not ULK1 or ULK2 knockout also promotes caspase-3 activation (Kaizuka and Mizushima, 2016), supporting a model in which MTFR1L controls the balance between autophagy and apoptosis through ATG13. Importantly, MG132 stabilization of ATG13 failed to rescue mitophagy, consistent with MTFR1L serving as a scaffold that links ATG13 to apoptotic regulation independently of ULK1.

By uncovering a reciprocal regulatory relationship between MTFR1L and the ULK1 complex, our study reveals how a fission factor can directly couple mitophagy to apoptosis. These findings position MTFR1L as a central determinant of mitochondrial quality control and cell fate, and open new directions for understanding how phosphorylation and cooperation with other fission factors fine-tune this balance.

## MATERIALS AND METHODS

### Cell culture and generation of stable cell lines

The mammalian plasmids and cell lines used in this study are listed in Table S4 and Table S5, respectively. The parental HEK293 (cCE26) and U2OS (cCE27, see Table S5) cell lines were obtained from Thermo Fisher Scientific (R78007 and K650001, respectively) and authenticated before use. Flp-In constructs were stably integrated into HEK293 and U2OS Flp-In T-REx cells, generating lines resistant to both hygromycin and blasticidin. Cells were maintained in Dulbecco's modified Eagle's medium (DMEM; Sigma-Aldrich, D6429) supplemented with 10% fetal bovine serum (FBS; Sigma-Aldrich, F7524-500ML), 5 U/ml penicillin and 50 µg/ml streptomycin (Sigma-Aldrich, P4333-100ML). Cultures were incubated at 37°C in a humidified atmosphere with 5% CO_2_. To generate stable Flp-In cell lines, 500,000 cells per well were seeded in six-well plates (Sarstedt, 83.3920) and cultured overnight in antibiotic-free medium. Cells were co-transfected with a 1:10 ratio of Flp-In expression vector (2×FKBP–GFP–ULK1, 2×FKBP–APEX2–ULK1, 2×FKBP–APEX2 or 2×FKBP–APEX2–ULK1-K46I) and the Flp recombinase plasmid pOG44 (Thermo Fisher Scientific, V600520). After 24 h, cells were placed in medium with 15 µg/ml blasticidin (InvivoGen, ant-bl-05). After an additional 24 h, cultures were expanded to 10-cm dishes (Sarstedt, 83.3902.300) and placed under dual selection using 15 µg/ml blasticidin and 100 µg/ml Hygromycin B Gold (InvivoGen, ant-hg-1) to select for integrants. The selection medium was replaced every 5 days. Individual colonies were manually picked, expanded in 24-well plates (Sarstedt, 83.3922.005) and screened for doxycycline-inducible expression of the integrated constructs by western blotting. All cell lines were routinely tested for mycoplasma contamination.

### siRNA treatment

For RNA interference experiments, U2OS cells were transfected with 20 nM siRNA using Lipofectamine RNAiMAX (Invitrogen) following the manufacturer's protocol. Transfections were performed for 72 h unless stated otherwise. To target MTFR1L, we used 5 different siRNAs. MTFR1L siRNA #1 was obtained from Integrated DNA Technologies (IDT; hs.Ri.MTFR1L.13.1). Additional MTFR1L siRNAs were obtained from Dharmacon Reagents as ON-TARGETplus Human MTFR1L siRNA (#2, J-017152-05; #3, J-017152-06; #4, J-017152-07; #5, J-017152-08). The siRNA targeting AMPKα1/2 was purchased from Santa Cruz Biotechnology (sc-45312). As a non-targeting control, ON-TARGETplus siRNA no. 1 (Dharmacon, D-001810-01-05) was used. For rescue experiments, only non-siRNA-resistant MTFR1L constructs were used. The use of a siRNA-resistant construct, in which silent mutations were introduced into the siRNA #1 target sequence of the *MTFR1L* coding region to confer siRNA resistance, revealed that overexpression of MTFR1L caused protein aggregation.

### Cell line transfection, lysis and immunoblot quantification

For transient transfections, cells were seeded in 10- or 15-cm dishes, six-well plates, or 35-mm glass-bottom dishes (ibidi, 81156) and cultured in antibiotic-free medium 24 h prior to transfection. Transfections were carried out using FuGENE HD (Promega) following the manufacturer's instructions. After 24 h, the medium was replaced with fresh complete growth medium and, where indicated, with specific treatments. Cells were harvested 48 h post transfection for immunoblotting, flow cytometry or microscopy. To induce bulk autophagy, cells were incubated in Earle's Balanced Salt Solution (EBSS; Sigma-Aldrich, E3024) for 4 h at 37°C. Following starvation, cells were washed once with Dulbecco's phosphate-buffered saline (DPBS; Sigma-Aldrich, D8537-500ML), detached using a cell scraper and collected in 15-ml conical tubes. Cell pellets were obtained by centrifugation at 500 ***g*** for 3 min and subsequently lysed in buffer containing 50 mM Tris-HCl (pH 8.0), 1 mM EDTA, 150 mM NaCl, 0.5% sodium deoxycholate, 0.1% SDS, 1% Triton X-100, 1 mM PMSF, 1 mM NaF, 20 mM β-glycerophosphate and cOmplete protease inhibitor cocktail (Roche). Lysates were clarified by centrifugation at 10,000 ***g*** for 15 min at 4°C. Protein concentrations were determined using the Pierce BCA Protein Assay kit (Thermo Fisher Scientific, 23227), and equal amounts of total protein were mixed with Laemmli sample buffer, boiled at 95°C for 5 min and resolved by SDS-PAGE. Immunoblot signals were quantified using FIJI. The band intensity of the protein of interest was normalized to that of the GAPDH loading control and further normalized to wild-type controls across three independent biological replicates. Uncropped blots are shown in Fig. S2.

### mt-mKeima assay

Cells were seeded in six-well plates (Sarstedt) at a density of 500,000 cells per well and cultured in DMEM supplemented with 10% FBS. After 24 h, cells were transiently transfected with 2×FKBP–GFP–ULK1 and, where indicated, with MTFR1L expression plasmid. Rapamycin analog (rapalog; A/C Heterodimerizer, TaKaRa, 635056) was added to the specified conditions at a final concentration of 0.5 µM for 24 h. Where indicated, Parkin-dependent mitophagy was induced by treatment with 4 µM antimycin A1 (Sigma-Aldrich, A8674) and 4 µM oligomycin (Sigma-Aldrich, 75351) (AO) for the indicated time points. For proteasomal inhibition, cells were treated with MG132 (Sigma-Aldrich, 474790) at a final concentration of 10 µM. Cells, where indicated, were additionally treated for 4 h with 200 nM bafilomycin A1 (Cell Signaling Technology, 54645) in the presence of rapalog. Cells were then harvested and washed with DPBS, detached using trypsin–EDTA (Sigma-Aldrich, T3924) and resuspended in FACS medium (Phenol Red-free DMEM, supplemented with 10% FBS). The suspension was transferred to 1.5 ml tubes and centrifuged at 500 ***g*** for 3 min at room temperature. Pellets were resuspended in 200 µl FACS medium and transferred to U-bottom 96-well plates (Greiner Bio-One, 650970) for flow cytometry analysis. Flow cytometry was performed using a CytoFLEX S flow cytometer (Beckman Coulter, B75408) and CytExpert v2.3 software (Beckman Coulter). mt-mKeima signal at neutral pH was excited with a 405 nm laser and at acidic pH with a 561 nm laser. Emission for both channels was detected using a 610/20 nm bandpass filter. GFP-positive cells were detected with a 488 nm laser and a 525/40 nm bandpass filter. A total of 100,000 events were collected per sample. Live cells were gated based on side scatter area (SSC-A) versus forward scatter area (FSC-A), and singlets were selected using forward scatter height (FSC-H) versus forward scatter width (FSC-W) gating. Analysis was restricted to GFP-positive events. Flow cytometry data were analyzed using FlowJo v10.9.0 (5 May 2023). Scale values of the selected populations were exported from FlowJo and processed using the Python library mKeima (v0.6.0; available at https://pypi.org/project/mkeima) for additional analysis.

### Phos-tag gel electrophoresis and phosphatase treatment

Phos-tag acrylamide (5 mM stock) was prepared according to the manufacturer's instructions (AAL-107, Wako Pure Chemical Industries). An 8% Phos-tag resolving gel (5 ml total volume; final concentrations: 20 µM Phos-tag, 100 µM ZnCl_2_) was prepared by combining 1.32 ml of 30% (w/v) acrylamide/0.8% (w/v) bisacrylamide (Rotiphorese Gel 30, Roth), 1.25 ml of 1.4 M BisTris-HCl (pH 6.8), 20 µl of 5 mM Phos-tag solution, 50 µl of freshly prepared 10 mM ZnCl_2_, 2.31 ml distilled H_2_O, 5 µl TEMED and 25 µl of 10% (w/v) ammonium persulfate (APS). The solution was gently mixed to minimize air bubble formation and poured immediately into gel cassettes. Distilled H_2_O was overlaid to ensure even polymerization. After polymerization, the water layer was removed and a 4% stacking gel (2.5 ml total volume) was cast by mixing 375 µl acrylamide solution, 625 µl of 1.4 M BisTris-HCl (pH 6.8), 1.49 ml H_2_O, 8.5 µl TEMED and 42.5 µl of 10% APS. Gels were polymerized fully prior to sample loading. For Phos-tag analysis, cells were scraped, pelleted at 5000 ***g*** for 4 min at 4°C, resuspended in 200 µl Tris-based lysis buffer [1×TBS; 0.1% Triton X-100, 1 mM dithiothreitol (DTT), 1 mM PMSF, 1 mM NaF, 1 mM Na_3_VO_4_ and cOmplete EDTA-free protease inhibitor cocktail (Roche)], and mechanically lysed by passing through a 26G needle 30 times. Lysates were clarified at 14,000 ***g*** for 15 min at 4°C, and protein concentrations were measured using the Bradford assay (Bio-Rad). Samples were adjusted to 2.5 µg/µl and mixed with 6× Laemmli sample buffer to 1× before boiling at 85°C for 5 min. Electrophoresis buffer (5 mM sodium bisulfite, 0.1 M Tris, 0.1 M MOPS and 0.1% SDS) was freshly prepared from 0.5 M sodium bisulfite stock and 5× running buffer (0.5 M Tris, 0.5 M MOPS and 0.5% SDS, pH 7.8). Gels were run at a constant current of 20 mA, and proteins were transferred to nitrocellulose membranes by wet electroblotting (Bio-Rad). For phosphatase treatment, cell lysates were prepared as described above but in NaF/Na_3_VO_4_-free lysis buffer. Dephosphorylation was carried out using λ protein phosphatase (New England Biolabs, P0753) at 30°C for 45 min with gentle agitation, following the manufacturer's protocol. The reaction was stopped by adding Laemmli sample buffer, and samples were boiled at 95°C for 5 min.

### Live-cell imaging

For microscopy, U2OS cells were seeded in 35-mm glass-bottom dishes (ibidi, 81156) and maintained in an environmental chamber at 37°C with 5% CO_2_ during image acquisition. Live-cell imaging was performed using a DeltaVision OMX Flex microscope (GE Healthcare) equipped with a UPlanSApo 60×/1.4 oil immersion Olympus objective and a PCO Edge 4.2 sCMOS camera. For inducible FKBP–FRB tethering, cells were treated with 0.5 µM rapalog for the indicated time. Mitochondria were stained with MitoTracker Deep Red FM (Invitrogen, M22426) according to the manufacturer's instructions prior to imaging.

### Mitochondrial network quantification

Mitochondrial morphology was quantified using MitoGraph (https://github.com/vianamp/MitoGraph; Viana et al., 2015). Images were acquired as described in the section above and analyzed as three-dimensional stacks. Preprocessing was performed with the recommended MitoGraph macros (CropCells and GenFramesMaxProjs), followed by conversion to 8-bit and background subtraction. Segmentation was performed using the default Otsu threshold, and mitochondrial networks were skeletonized with default pruning. Multiple parameters were extracted per cell, e.g. total network length, number of nodes and edges. The ‘average edge length’ reported corresponds to the mean segment length between nodes per cell, expressed in micrometers. Cells with saturated signal, motion blur or failed segmentation were excluded prior to analysis.

### Cell viability assay

To assess cell viability following nutrient deprivation and siRNA treatment, a Crystal Violet staining assay was performed. Optimal seeding densities for each condition were determined in preliminary experiments to ensure comparable confluency. Based on these findings, cells were seeded in six-well plates (Sarstedt, 83.3920) at a density of 500,000 cells per well for siControl and 1,000,000 cells per well for siMTFR1L conditions. Cells were maintained in DMEM supplemented with 10% FBS and 5 U/ml penicillin and 50 µg/ml streptomycin. After 24 h, cells were transfected with 20 nM siRNA targeting *MTFR1L* or non-targeting control siRNA as previously described. To induce bulk autophagy, cells were treated for 4 h with either complete growth medium, EBSS supplemented with 1 µM Torin 1 (Tocris, 4247), or EBSS supplemented with 1 µM Torin 1 and 1 µM Emricasan (Selleck Chemicals, S7775). Following treatment, cells were washed twice with DPBS and fixed in 100% methanol at −20°C for 20 min (0.5 ml per well). The methanol was discarded and cells were washed twice with DPBS before staining. Fixed cells were stained with 0.5% (w/v) Crystal Violet (Sigma-Aldrich, C0775) in distilled water for 30 min at room temperature. Plates were then submerged three times in distilled water to remove excess stain and dried overnight at room temperature. Twenty randomly selected fields per well were imaged using bright-field microscopy with a 20× objective. Live cells were defined by the presence of an intact nucleus, uniform but not excessive Crystal Violet staining, preserved cellular shape and tonus, absence of shrinkage, and intact plasma membrane. Cells were excluded from live counts if they exhibited nuclear fragmentation, homogeneous and abnormally intense staining, or altered morphology. Manual quantification was performed using the Cell Counter plugin in FIJI. For analysis, live-cell counts from six fields per replicate were averaged and normalized to non-starved control samples, which were set as 100% cell survival. Relative survival rates were then calculated for each treatment condition.

### Oxygen consumption rate measurements

To compare mitochondrial performance between wild-type and MTFR1L-depleted cells, cells were transfected with siControl or siMTFR1L for 72 h as described above. Cells were then seeded into Agilent Seahorse XF cell culture microplates at a density of 50,000 cells per well and allowed to adhere for 6 h. Four hours before the assay, cells were washed and incubated with EBSS to induce starvation at 37°C. The Agilent Seahorse XF Cell Mito Stress Test protocol was followed according to the manufacturer's instructions. In brief, the following compounds were sequentially injected into each well at the indicated final concentrations: 1.5 µM oligomycin (Sigma-Aldrich, O4876), 2 µM carbonyl cyanide-4-(trifluoromethoxy)phenylhydrazone (FCCP) (Sigma-Aldrich, C2920) and 0.5 µM rotenone/antimycin A (Sigma-Aldrich, R8875 and A8674, respectively). The oxygen consumption rate (OCR) was measured using the Agilent Seahorse XF analyzer. OCR values were normalized to cell numbers.

### Protein purification

GST and GST fusion proteins were expressed in BL21(DE3) cells (69450, Novagen). A single colony of transformed *Escherichia coli* BL21(DE3) cells expressing GST-tagged constructs (listed in Table S6) was inoculated into 5 ml of LB medium supplemented with 100 µg/ml ampicillin and cultured overnight at 37°C with shaking. The overnight culture was transferred into 250 ml of fresh LB medium containing ampicillin and incubated at 37°C until the optical density at 600 nm (OD_600_) reached 0.7–1.0. Protein expression was induced by adding 0.5 mM IPTG (Thermo Fisher Scientific), followed by overnight incubation at 16°C. Cells were harvested by centrifugation at 4000 ***g*** for 10 min at 4°C. Cell pellets were resuspended in GST lysis buffer (50 mM Tris-HCl pH 8.0, 150 mM NaCl, 1 mM DTT, 1 mM PMSF and cOmplete protease inhibitor cocktail) at one to two times the pellet volume. Lysis was performed by sonication at 20% amplitude on ice using three cycles of 20 s ON with 30 s OFF intervals. Lysates were clarified by centrifugation at 14,000 ***g*** for 15 min at 4°C, and the supernatant containing soluble protein was transferred to fresh tubes. For affinity purification, 60 µl of glutathione sepharose 4B beads (Cytiva) per sample was equilibrated in GST lysis buffer and pelleted by centrifugation at 500 ***g*** for 1 min. Clarified lysate was added to the beads and incubated at 4°C for 1 h with gentle tumbling. After binding, the beads were washed three times with GST lysis buffer to remove unbound proteins. Elution was carried out by adding 200 µl of GST elution buffer (50 mM Tris-HCl pH 8.0, 20 mM reduced glutathione) and incubating at room temperature for 20 min. This step was repeated two times and, after each elution, the sample was centrifuged at 500 ***g*** for 1 min at 4°C. Supernatants containing the eluted protein were transferred to clean tubes. Eluted protein samples were buffer-exchanged into 1×PBS and concentrated using centrifugal filter units (Amicon Ultra, 10 kDa MWCO) to achieve uniform protein concentrations. Protein isolation was verified by SDS-PAGE and Coomassie Brilliant Blue staining. Purified proteins were stored at −20°C for downstream applications.

### *In vitro* phosphorylation

Per phosphorylation reaction, 3 µg (3 µg/µl) of isolated GST-tagged substrate was incubated with 0.1 µg purchased recombinant ULK1, purified from FreeStyle 293-F cells (Sigma-Aldrich, SRP0252), and 1 µCi γ-[^32^P]-ATP (PerkinElmer) in a 11 µl reaction volume. The phosphorylation reaction was carried out at 30°C for 30 min in kinase reaction buffer containing 20 mM HEPES (pH 7.4), 150 mM potassium acetate, 10 mM magnesium acetate, 0.5 mM EGTA, 5 mM NaCl and 10 mM Na_3_VO_4_. The reaction was terminated by adding 3× urea loading buffer to a final concentration of 1×, and samples were resolved by SDS-PAGE with the addition of polyacrylamide (final concentration of 0.35%) to stabilize the gel during the subsequent drying process. Phosphorylated proteins were detected by phosphor imaging using a Typhoon scanner (Cytiva).

### Sample preparation for MS analysis

HEK293 Flp-In T-REx cells were freshly thawed and seeded. The day before collection, cells were treated with 1 µg/ml doxycycline (Sigma-Aldrich, D9891-10g) to induce expression of the Flp-In integrated constructs: 2×FKBP–myc–APEX2 for cRB12 cells (see Table S5), 2×FKBP–myc–APEX2–ULK1 for cRB7 cells (Table S5) and 2×FKBP–myc–APEX2–ULK1-K46I for cRB20 cells (Table S5). On the day of collection, cells were treated with 500 µM biotin–phenol (IrisBiotech, 41994-02-9/LS-3500.5000) and, where indicated, with 0.5 µM rapalog for 1 h at 37°C. To induce peroxidase activity of APEX2 and formation of biotinylated proteins, cells were treated for 1 min with 1 mM H_2_O_2_ (Roth, CP26.5). Immediately after the 1 min H_2_O_2_ pulse, cells were washed with DPBS and subsequently washed with quenching buffer [DPBS containing 10 mM sodium ascorbate (Sigma-Aldrich, 11140-50G) and 5 mM Trolox (Sigma-Aldrich, 238813-25G)] to prevent further biotinylation. Cells were scraped, collected and lysed in RIPA buffer [50 mM Tris, 150 mM NaCl, 0.1% SDS, 0.5% sodium deoxycholate, 1% Triton X-100 and 1× protease inhibitor cocktail (Roche, 05056489001)] supplemented with quenching reagents (10 mM sodium ascorbate and 1 mM Trolox). Lysates were clarified by centrifugation at 10,000 ***g*** for 15 min at 4°C. Magnetic streptavidin beads (Pierce, Thermo Fisher Scientific, 88816) were chemically acetylated using Sulfo-NHS-Acetate (Pierce, Thermo Fisher Scientific, 26777) to prevent contamination with co-digested streptavidin peptides (Hollenstein et al., 2023). The supernatant was incubated for 1 h at 4°C with the Sulfo-NHS-Acetate-treated magnetic streptavidin beads. After incubation, samples were washed five times with Tris buffer (50 mM Tris and 150 mM NaCl) and three times with 50 mM ammonium bicarbonate buffer (Sigma-Aldrich, 09830-1KG). The beads were transferred to a new tube and resuspended in 50 µl of 1 M urea and 50 mM ammonium bicarbonate. Samples were reduced with 2 µl of 250 mM DTT (Roche, 10708984001) for 30 min at room temperature and alkylated with 2 µl of 500 mM iodoacetamide (Sigma-Aldrich, I6125-5G) for 30 min at room temperature in the dark. The remaining iodoacetamide was quenched with 1 µl of 250 mM DTT for 10 min. Proteins were digested with 150 ng LysC (FUJIFILM Wako Pure Chemical, 125-02543) at 25°C overnight. The supernatant was transferred to a new 0.2-ml vial and further digested for 5 h at 37°C by addition of 150 ng trypsin (Trypsin Gold, MS grade, Promega, V5280). The digest was stopped by addition of trifluoroacetic acid to a final concentration of 0.5%, and peptides were desalted using a 96-well OASIS HLB µElution plate (Waters, 30-µm particle size, 186001828BA) following the manufacturer's protocol.

### Liquid chromatography–MS analysis

Liquid chromatography (LC)–MS analysis was performed on an UltiMate 3000 RSLCnano LC system (Thermo Fisher Scientific) coupled to an Orbitrap Exploris 480 mass spectrometer (Thermo Fisher Scientific). The system was equipped with a high-field asymmetric-waveform ion mobility spectrometry (FAIMS) Pro interface (Thermo Fisher Scientific), a Nanospray Flex ion source (Thermo Fisher Scientific), coated emitter tips (PepSep, MSWil) and a Butterfly Portfolio Heater (Phoenix S&T). Peptides were loaded onto a trap column (PepMap Neo C18 5 mm×300 µm, 5 μm particle size, Thermo Fisher Scientific) using 0.1% trifluoroacetic acid as mobile phase, and separated on an analytical column (Acclaim PepMap 100 C18 HPLC Column, 50 cm×75 µm, 2 μm particle size, Thermo Fisher Scientific), applying a linear gradient starting with a mobile phase of 98% solvent A (0.1% formic acid) and 2% solvent B (80% acetonitrile, 0.08% formic acid), increasing to 35% solvent B over 60 min at a flow rate of 230 nl/min. The analytical column was heated to 30°C. The mass spectrometer was operated in data-independent acquisition (DIA) mode with FAIMS compensation voltage set to −45 V, with a 2.5 s cycle time. Survey scans were acquired from a mass to charge ratio (*m/z*) of 350–1200, normalized automatic gain control (AGC) target of 300%, and resolution of 60,000. Per cycle, 31 MS2 spectra were acquired across a mass range of 349.5–1200.5 *m/z* using variable isolation windows (13–257 *m/z*) with 1 *m/z* overlap between windows. Selected ions were analyzed using automatic maximum injection time, normalized AGC target of 1000% and resolution of 30,000 after higher-energy collisional dissociation (HCD) fragmentation with normalized collision energy of 30%.

### MS data analysis

MS raw data were processed with Spectronaut (18.6, Biognosys). The library-free DirectDIA+ workflow was used for analysis of the raw files, the *Homo sapiens* one protein per gene reference proteome from Uniprot (proteome ID: UP000005640, release 2023.03), concatenated with a database of 379 common laboratory contaminants (release 2023.01, https://github.com/maxperutzlabs-ms/perutz-ms-contaminants) and an entry for the 2×FKBP–APEX2 construct. The cleavage specificity was set to full trypsin specificity (Trypsin/P), with two missed cleavages allowed. Carbamidomethyl was used as fixed cysteine modification; methionine oxidation and protein N-terminal acetylation were specified as variable modifications. The thresholds for precursor q-value, precursor posterior error probability (PEP), protein q-value per experiment, protein q-value per run and protein PEP were all set at 1% (Baker et al., 2024). Cross-run normalization was disabled, and all other settings were used at their default values. Computational analysis was performed using Python and the in-house developed Python library MsReport (version 0.0.23; source code: https://github.com/hollenstein/msreport; archive: doi:10.5281/zenodo.15309090).

Only non-contaminant proteins identified with a minimum of two peptides and being quantified in at least two replicates of one condition were considered for further analysis. Proteins with an intensity below 1000 were removed and treated as not quantified to exclude low-quality quantification. MS2 label-free quantification (LFQ) protein intensities were log2-transformed and normalized across samples using ModeNormalizer from MsReport. The ModeNormalizer method involves calculating log2 protein ratios for all pairs of samples and determining normalization factors based on the modes of all ratio distributions. Missing values were imputed by drawing random values from a normal distribution with mean (μ)=9.96 and standard deviation (σ)=0.75. intensity-based absolute quantification (iBAQ) intensities were calculated by dividing protein intensities by the number of theoretically observable tryptic peptides between six and 30 amino acids. Statistical analysis was performed using the linear models for microarray analysis (limma) v.3.54.2 (Ritchie et al., 2015) package in R. Moderated *t*-statistics were calculated using the limma-trend method, and multiple testing correction was applied using the Benjamini–Hochberg method. The results are summarized in Table S1 (data used for Fig. 1D).

ULK1-specific proteins were selected based on an adjusted *P*-value<0.01 and log2 ratio>1 in the FKBP–APEX2–ULK1 (WT) versus FKBP–APEX2 (WT) conditions, and the FKBP–APEX2–ULK1-K46I (WT) versus FKBP–APEX2 (WT) conditions. Average log2 protein intensities were calculated for the FKBP–APEX2 (WT) condition. The mean of the controls was subtracted from each replicate of the respective FKBP–APEX2–ULK1 or FKBP–APEX–ULK1-K46I samples, resulting in values denoted as ‘signal over control (log2)’. Moderated *t*-statistics were calculated using limma without the trend option, and multiple testing correction was applied using the Benjamini–Hochberg method. The results are summarized in Table S2 (data used for Fig. 1C).

Gene Ontology terms were obtained from UniProt (release 2023.05). The Python library XlsxReport (version 0.0.5; source code: https://github.com/hollenstein/xlsxreport; archive: doi:10.5281/zenodo.15129818) was used to create formatted Excel files summarizing the results of the proteomics experiments.

A subset of the proteomics mass spectrometry raw data was previously published in Licheva et al. (2025) and deposited to the ProteomeXchange Consortium via the PRIDE (Perez-Riverol et al., 2022) partner repository with the dataset identifier PXD047277. The full dataset was reanalyzed in this study and the mass spectrometry proteomics data have been deposited to the ProteomeXchange Consortium with the dataset identifier PXD068381.

### Antibodies

Rabbit monoclonal anti-ULK1 (#8054), rabbit monoclonal anti-FIP200 (#12436), rabbit monoclonal anti-ATG13 (D4P1K, #13273), rabbit monoclonal anti-ATG101 (E1Z4W, #13492), rabbit monoclonal anti-phospho-ATG14 (Ser29) (D4B8M, #92340), rabbit polyclonal anti-PARP (#9542), rabbit polyclonal anti-caspase-3 (#9662), rabbit monoclonal anti-AMPK (#2532), rabbit monoclonal anti-α-tubulin (#2144) and rabbit monoclonal anti-GAPDH (14C10, #2118) were purchased from Cell Signaling Technology and used at 1:1000 dilution. Rabbit polyclonal anti-MTFR1L (HPA027124) was purchased from Sigma-Aldrich and used at 1:1000. Mouse monoclonal anti-Parkin (sc-32282) was obtained from Santa Cruz Biotechnology and used at 1:1000. Rabbit monoclonal anti-vinculin (#700062) and rabbit polyclonal anti-FKBP12 (PA1-026A) were obtained from Thermo Fisher Scientific and used at 1:1000. Mouse monoclonal anti-mKeima (M126-3M) was obtained from MBL Life Science and used at 1:1000. For secondary antibodies, goat anti-rabbit IgG (H+L) HRP conjugate (#1706515) and goat anti-mouse IgG (H+L) HRP conjugate (#1706516) were obtained from Bio-Rad and used at 1:10,000.

### Statistical analysis and reproducibility

At least three independent biological replicates were performed for each experiment, except for Figs 3D and 5B, for which two replicates were conducted. No data were excluded from analysis except in cases of clear technical failure. Statistical significance was assessed using a one-way ANOVA followed by Sidak's multiple comparisons test, as specified in the figure legends. In all figures, statistical significance is indicated as follows: n.s., not significant; *P<0.05; **P<0.01; ***P<0.001; ****P<0.0001. Data distribution was assumed to be normal, although this was not formally tested. For all fluorescence microscopy experiments, cells were randomly selected from the brightfield channel without bias toward the fluorescence signal. Fluorescence quantification was performed manually after randomizing file names to avoid experimenter bias. No statistical methods were used to predetermine sample sizes, but our sample sizes are comparable to those reported in previous studies. Source data including exact sample sizes, individual data points and statistical test results for all quantified figures are provided in Table S3.

### AI disclosure statement

Artificial intelligence (AI)-based language tools (ChatGPT and Google Gemini) were used solely to assist with wording and language refinement. These tools were not used to generate scientific content, interpret results or draw conclusions. After using these services, the authors reviewed, verified and edited the content as needed and take full responsibility for the content of the publication.

## Supplementary Material



10.1242/joces.264577_sup1 Supplementary information

Table S1. Mass spectrometry data for APEX2-based proximity labeling

Table S2. Signal-over-control analysis of ULK1-specific proteins

Table S3. Source data for quantified figures

## References

[JCS264577C1] Allen, G. F. G., Toth, R., James, J. and Ganley, I. G. (2013). Loss of iron triggers PINK1/Parkin-independent mitophagy. *EMBO Rep.* 14, 1127-1135. 10.1038/embor.2013.168 24176932 PMC3981094

[JCS264577C2] Baker, C. P., Bruderer, R., Abbott, J., Arthur, J. S. C. and Brenes, A. J. (2024). Optimizing spectronaut search parameters to improve data quality with minimal proteome coverage reductions in DIA analyses of heterogeneous samples. *J. Proteome Res.* 23, 1926-1936. 10.1021/acs.jproteome.3c00671 38691771 PMC11165578

[JCS264577C3] Banaszynski, L. A., Liu, C. W. and Wandless, T. J. (2006). Characterization of the FKBP·rapamycin·FRB ternary complex [J. Am. Chem. Soc. 2005, 127, 4715–4721]. *J. Am. Chem. Soc.* 128, 15928. 10.1021/ja0699788 15796538

[JCS264577C4] Bellot, G., Garcia-Medina, R., Gounon, P., Chiche, J., Roux, D., Pouysségur, J. and Mazure, N. M. (2009). Hypoxia-induced autophagy is mediated through hypoxia-inducible factor induction of BNIP3 and BNIP3L via their BH3 domains. *Mol. Cell. Biol.* 29, 2570-2581. 10.1128/MCB.00166-09 19273585 PMC2682037

[JCS264577C5] Chen, Z., Lei, C., Wang, C., Li, N., Srivastava, M., Tang, M., Zhang, H., Choi, J. M., Jung, S. Y., Qin, J. et al. (2019). Global phosphoproteomic analysis reveals ARMC10 as an AMPK substrate that regulates mitochondrial dynamics. *Nat. Commun.* 10, 104. 10.1038/s41467-018-08004-0 30631047 PMC6328551

[JCS264577C6] Egan, D. F., Chun, M. G. H., Vamos, M., Zou, H., Rong, J., Miller, C. J., Lou, H. J., Raveendra-Panickar, D., Yang, C.-C., Sheffler, D. J. et al. (2015). Small molecule inhibition of the autophagy kinase ULK1 and identification of ULK1 substrates. *Mol. Cell* 59, 285-297. 10.1016/j.molcel.2015.05.031 26118643 PMC4530630

[JCS264577C7] Eickhorst, C., Babic, R., Rush-Kittle, J., Lucya, L., Imam, F. L., Sánchez-Martín, P., Hollenstein, D. M., Michaelis, J., Münch, C., Meisinger, C. et al. (2024). FIP200 phosphorylation regulates late steps in mitophagy. *J. Mol. Biol.* 436, 168631. 10.1016/j.jmb.2024.168631 38821350

[JCS264577C8] Ganley, I. G. and Simonsen, A. (2022). Diversity of mitophagy pathways at a glance. *J. Cell Sci.* 135, jcs259748. 10.1242/jcs.259748 36504076 PMC10656428

[JCS264577C9] Heo, J.-M., Ordureau, A., Paulo, J. A., Rinehart, J. and Harper, J. W. (2015). The PINK1-PARKIN mitochondrial ubiquitylation pathway drives a program of OPTN/NDP52 recruitment and TBK1 activation to promote mitophagy. *Mol. Cell* 60, 7-20. 10.1016/j.molcel.2015.08.016 26365381 PMC4592482

[JCS264577C10] Hollenstein, D. M., Maurer-Granofszky, M., Reiter, W., Anrather, D., Gossenreiter, T., Babic, R., Hartl, N., Kraft, C. and Hartl, M. (2023). Chemical acetylation of ligands and two-step digestion protocol for reducing codigestion in affinity purification-mass spectrometry. *J. Proteome Res.* 22, 3383-3391. 10.1021/acs.jproteome.3c00424 37712406 PMC10563155

[JCS264577C11] Kaizuka, T. and Mizushima, N. (2016). Atg13 is essential for autophagy and cardiac development in mice. *Mol. Cell. Biol.* 36, 585-595. 10.1128/MCB.01005-15 26644405 PMC4751695

[JCS264577C12] Katayama, H., Kogure, T., Mizushima, N., Yoshimori, T. and Miyawaki, A. (2011). A sensitive and quantitative technique for detecting autophagic events based on lysosomal delivery. *Chem. Biol.* 18, 1042-1052. 10.1016/j.chembiol.2011.05.013 21867919

[JCS264577C13] Lazarou, M., Sliter, D. A., Kane, L. A., Sarraf, S. A., Wang, C., Burman, J. L., Sideris, D. P., Fogel, A. I. and Youle, R. J. (2015). The ubiquitin kinase PINK1 recruits autophagy receptors to induce mitophagy. *Nature* 524, 309-314. 10.1038/nature14893 26266977 PMC5018156

[JCS264577C14] Licheva, M., Pflaum, J., Babic, R., Mancilla, H., Elsässer, J., Boyle, E., Hollenstein, D. M., Jimenez-Niebla, J., Pleyer, J., Heinrich, M. et al. (2025). Phase separation of initiation hubs on cargo is a trigger switch for selective autophagy. *Nat. Cell Biol.* 27, 283-297. 10.1038/s41556-024-01572-y 39774270 PMC11821514

[JCS264577C15] Matsuda, N., Sato, S., Shiba, K., Okatsu, K., Saisho, K., Gautier, C. A., Sou, Y.-S., Saiki, S., Kawajiri, S., Sato, F. et al. (2010). PINK1 stabilized by mitochondrial depolarization recruits Parkin to damaged mitochondria and activates latent Parkin for mitophagy. *J. Cell Biol.* 189, 211-221. 10.1083/jcb.200910140 20404107 PMC2856912

[JCS264577C16] Mercer, T. J., Ohashi, Y., Boeing, S., Jefferies, H. B. J., De Tito, S., Flynn, H., Tremel, S., Zhang, W., Wirth, M., Frith, D. et al. (2021). Phosphoproteomic identification of ULK substrates reveals VPS15-dependent ULK/VPS34 interplay in the regulation of autophagy. *EMBO J.* 40, e105985. 10.15252/embj.2020105985 34121209 PMC8280838

[JCS264577C17] Munson, M. J., Mathai, B. J., Ng, M. Y. W., Trachsel-Moncho, L., De La Ballina, L. R., Schultz, S. W., Aman, Y., Lystad, A. H., Singh, S., Singh, S. et al. (2021). GAK and PRKCD are positive regulators of PRKN-independent mitophagy. *Nat. Commun.* 12, 6101. 10.1038/s41467-021-26331-7 34671015 PMC8528926

[JCS264577C18] Narendra, D., Tanaka, A., Suen, D.-F. and Youle, R. J. (2008). Parkin is recruited selectively to impaired mitochondria and promotes their autophagy. *J. Cell Biol.* 183, 795-803. 10.1083/jcb.200809125 19029340 PMC2592826

[JCS264577C19] Narendra, D. P., Jin, S. M., Tanaka, A., Suen, D.-F., Gautier, C. A., Shen, J., Cookson, M. R. and Youle, R. J. (2010). PINK1 is selectively stabilized on impaired mitochondria to activate Parkin. *PLoS Biol.* 8, e1000298. 10.1371/journal.pbio.1000298 20126261 PMC2811155

[JCS264577C20] Onishi, M., Yamano, K., Sato, M., Matsuda, N. and Okamoto, K. (2021). Molecular mechanisms and physiological functions of mitophagy. *EMBO J.* 40, e104705. 10.15252/embj.2020104705 33438778 PMC7849173

[JCS264577C21] Papinski, D., Schuschnig, M., Reiter, W., Wilhelm, L., Barnes, C. A., Maiolica, A., Hansmann, I., Pfaffenwimmer, T., Kijanska, M., Stoffel, I. et al. (2014). Early steps in autophagy depend on direct phosphorylation of Atg9 by the Atg1 kinase. *Mol. Cell* 53, 471-483. 10.1016/j.molcel.2013.12.011 24440502 PMC3978657

[JCS264577C22] Park, J.-M., Jung, C. H., Seo, M., Otto, N. M., Grunwald, D., Kim, K. H., Moriarity, B., Kim, Y.-M., Starker, C., Nho, R. S. et al. (2016). The ULK1 complex mediates MTORC1 signaling to the autophagy initiation machinery via binding and phosphorylating ATG14. *Autophagy* 12, 547-564. 10.1080/15548627.2016.1140293 27046250 PMC4835982

[JCS264577C23] Park, J.-M., Lee, D.-H. and Kim, D.-H. (2023). Redefining the role of AMPK in autophagy and the energy stress response. *Nat. Commun.* 14, 2994. 10.1038/s41467-023-38401-z 37225695 PMC10209092

[JCS264577C24] Perez-Riverol, Y., Bai, J., Bandla, C., García-Seisdedos, D., Hewapathirana, S., Kamatchinathan, S., Kundu, D. J., Prakash, A., Frericks-Zipper, A., Eisenacher, M. et al. (2022). The PRIDE database resources in 2022: a hub for mass spectrometry-based proteomics evidences. *Nucleic Acids Res.* 50, D543-D552. 10.1093/nar/gkab1038 34723319 PMC8728295

[JCS264577C25] Richter, B., Sliter, D. A., Herhaus, L., Stolz, A., Wang, C., Beli, P., Zaffagnini, G., Wild, P., Martens, S., Wagner, S. A. et al. (2016). Phosphorylation of OPTN by TBK1 enhances its binding to Ub chains and promotes selective autophagy of damaged mitochondria. *Proc. Natl Acad. Sci. USA* 113, 4039-4044. 10.1073/pnas.1523926113 27035970 PMC4839414

[JCS264577C26] Ritchie, M. E., Phipson, B., Wu, D., Hu, Y., Law, C. W., Shi, W. and Smyth, G. K. (2015). limma powers differential expression analyses for RNA-sequencing and microarray studies. *Nucleic Acids Res.* 43, e47. 10.1093/nar/gkv007 25605792 PMC4402510

[JCS264577C27] Schaffer, B. E., Levin, R. S., Hertz, N. T., Maures, T. J., Schoof, M. L., Hollstein, P. E., Benayoun, B. A., Banko, M. R., Shaw, R. J., Shokat, K. M. et al. (2015). Identification of AMPK phosphorylation sites reveals a network of proteins involved in cell invasion and facilitates large-scale substrate prediction. *Cell Metab.* 22, 907-921. 10.1016/j.cmet.2015.09.009 26456332 PMC4635044

[JCS264577C28] Shi, L., Sun, Z., Cao, Y., Li, Y., Qing, W., Zhou, L., Xu, M., Mai, X., Ou, L., Yang, X. et al. (2026). MTFR1L is a cardiac antiaging factor for maintenance of mitochondrial homeostasis. *Proc. Natl. Acad. Sci. USA* 123, e2527247123. 10.1073/pnas.2527247123 41576098 PMC12846826

[JCS264577C29] Tanaka, A., Cleland, M. M., Xu, S., Narendra, D. P., Suen, D.-F., Karbowski, M. and Youle, R. J. (2010). Proteasome and p97 mediate mitophagy and degradation of mitofusins induced by Parkin. *J. Cell Biol.* 191, 1367-1380. 10.1083/jcb.201007013 21173115 PMC3010068

[JCS264577C30] Tilokani, L., Russell, F. M., Hamilton, S., Virga, D. M., Segawa, M., Paupe, V., Gruszczyk, A. V., Protasoni, M., Tabara, L.-C., Johnson, M. et al. (2022). AMPK-dependent phosphorylation of MTFR1L regulates mitochondrial morphology. *Sci. Adv.* 8, eabo7956. 10.1126/sciadv.abo7956 36367943 PMC9651865

[JCS264577C31] Torggler, R., Papinski, D., Brach, T., Bas, L., Schuschnig, M., Pfaffenwimmer, T., Rohringer, S., Matzhold, T., Schweida, D., Brezovich, A. et al. (2016). Two independent pathways within selective autophagy converge to activate Atg1 kinase at the vacuole. *Mol. Cell* 64, 221-235. 10.1016/j.molcel.2016.09.008 27768871

[JCS264577C32] Tracy, K., Dibling, B. C., Spike, B. T., Knabb, J. R., Schumacker, P. and Macleod, K. F. (2007). BNIP3 is an RB/E2F target gene required for hypoxia-induced autophagy. *Mol. Cell. Biol.* 27, 6229-6242. 10.1128/MCB.02246-06 17576813 PMC1952167

[JCS264577C33] Vargas, J. N. S., Wang, C., Bunker, E., Hao, L., Maric, D., Schiavo, G., Randow, F. and Youle, R. J. (2019). Spatiotemporal control of ULK1 activation by NDP52 and TBK1 during selective autophagy. *Mol. Cell* 74, 347-362.e6. 10.1016/j.molcel.2019.02.010 30853401 PMC6642318

[JCS264577C34] Viana, M. P., Lim, S. and Rafelski, S. M. (2015). Quantifying mitochondrial content in living cells. *Methods Cell Biol.* 125, 77-93. 10.1016/bs.mcb.2014.10.003 25640425

[JCS264577C35] Wold, M. S., Lim, J., Lachance, V., Deng, Z. and Yue, Z. (2016). ULK1-mediated phosphorylation of ATG14 promotes autophagy and is impaired in Huntington's disease models. *Mol. Neurodegener.* 11, 76. 10.1186/s13024-016-0141-0 27938392 PMC5148922

[JCS264577C36] Yamashita, S.-I., Jin, X., Furukawa, K., Hamasaki, M., Nezu, A., Otera, H., Saigusa, T., Yoshimori, T., Sakai, Y., Mihara, K. et al. (2016). Mitochondrial division occurs concurrently with autophagosome formation but independently of Drp1 during mitophagy. *J. Cell Biol.* 215, 649-665. 10.1083/jcb.201605093 27903607 PMC5147001

