# Supplementary material for: A ULK1–MTFR1L feedback loop links mitochondrial fission, mitophagy and apoptosis
Source: J Cell Sci. 2026 May 11;139(9):jcs264577. doi: 10.1242/jcs.264577 (PMC13245916; doi:10.1242/jcs.264577)
Supplement: Supplementary information [file joces-139-264577-s1.pdf]

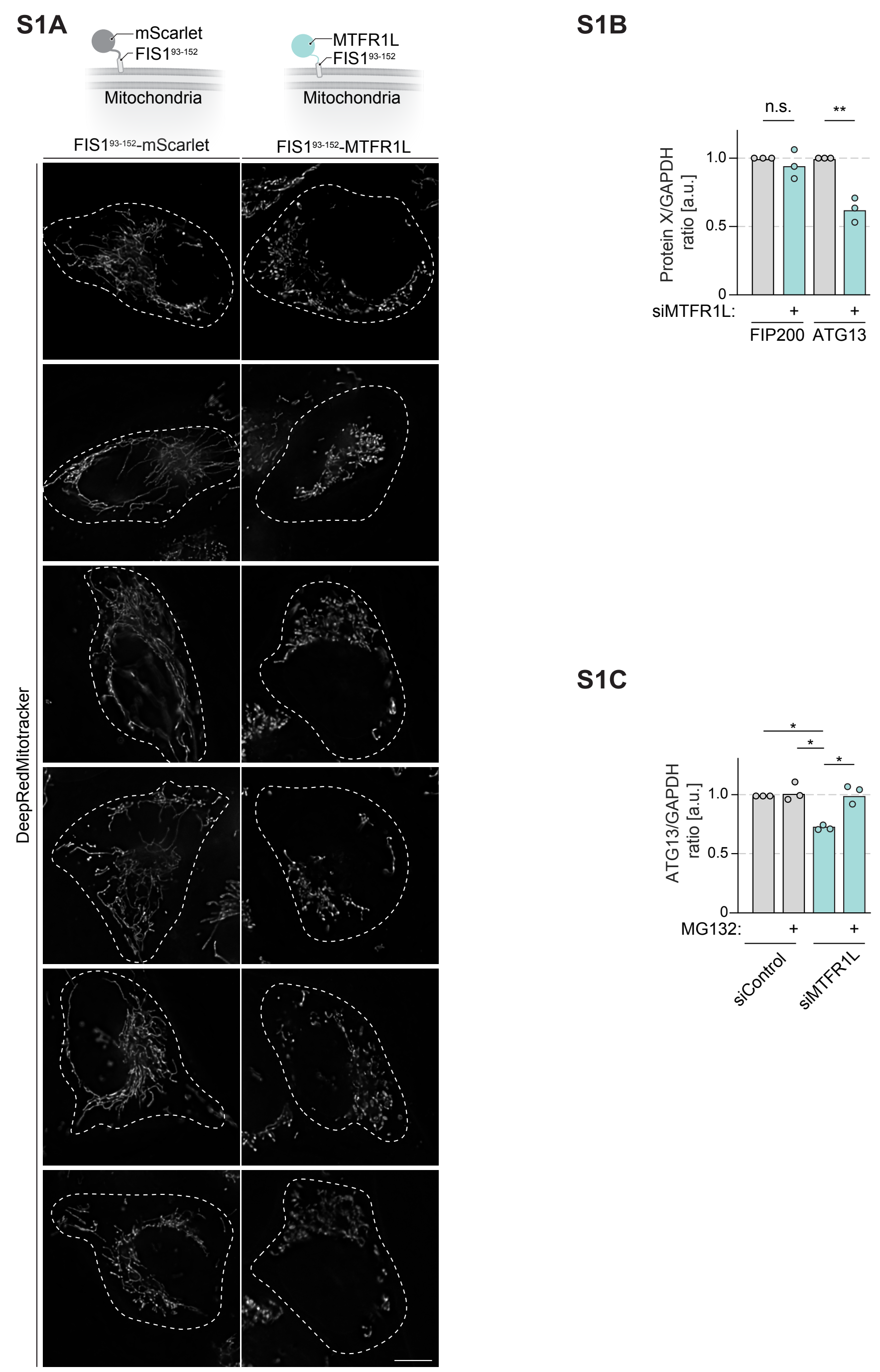

Fig. S1. Related to Fig. 1E, 3G and 3H

A) Additional representative images of U2OS cells (cCE27) transiently transfected with FIS1$^{93-152}$-mScarlet or FIS1$^{93-152}$-MTFR1L, stained with MitoTrackerDeepRed. Images complement Figure 1E and show further examples of mitochondrial network morphology across conditions. Scale bar: 10 µm.

B) Quantification of FIP200/GAPDH and ATG13/GAPDH ratios from Figure 3G. U2OS cells

Journal of Cell Science • Supplementary information

(cCE377) stably expressing FRB-FIS1$^{93\text{-}152}$, mt-mKeima and 2xFKBP-GFP-ULK1 were treated with control or #1 MTFR1L siRNA for 72 h. Band intensities were quantified from three independent biological replicates. Each circle represents the mean value of one biological replicate. Bars show the mean of three independent biological replicates. Statistical analysis: one-way ANOVA followed by Sidak's multiple comparison test.

C) Quantification of ATG13/GAPDH ratios from Figure 3H. U2OS cells (cCE377) stably expressing FRB-FIS1$^{93\text{-}152}$, mt-mKeima and 2xFKBP-GFP-ULK1 were treated with control or #1 MTFR1L siRNA for 72 h. Where indicated, cells were treated with 10 µM MG132 for 4 h. Band intensities were quantified from three independent biological replicates. Each circle represents the mean value of one biological replicate. Bars show the mean of three independent biological replicates. Statistical analysis: one-way ANOVA followed by Sidak's multiple comparison test.

Journal of Cell Science • Supplementary information

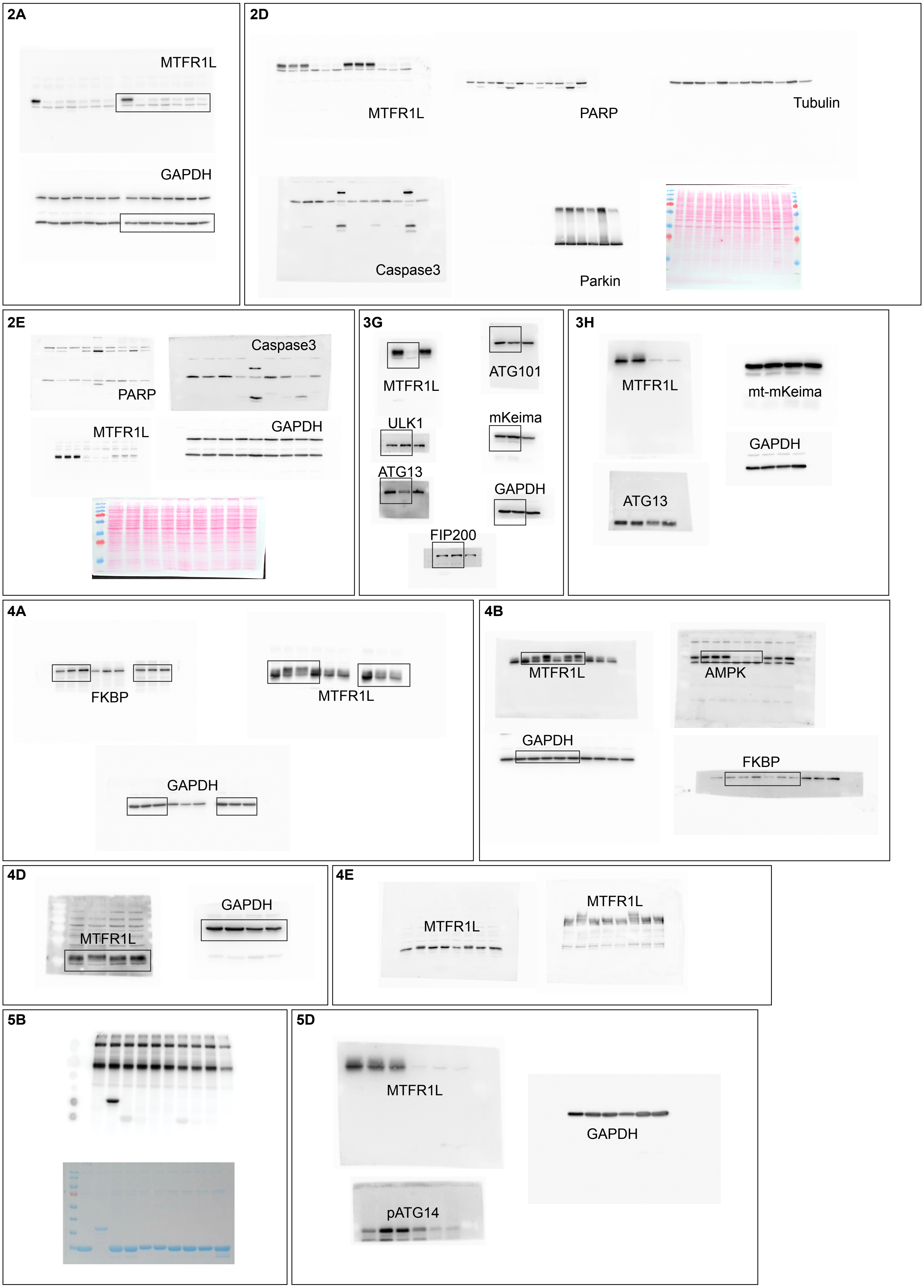

Fig. S2. Uncropped western blot images

Journal of Cell Science • Supplementary information

Table S1. Mass spectrometry data for APEX2-based proximity labeling

Available for download at
https://journals.biologists.com/jcs/article-lookup/doi/10.1242/jcs.264577#supplementary-data

Table S2. Signal-over-control analysis of ULK1-specific proteins

Available for download at
https://journals.biologists.com/jcs/article-lookup/doi/10.1242/jcs.264577#supplementary-data

Table S3. Source data for quantified figures

Available for download at
https://journals.biologists.com/jcs/article-lookup/doi/10.1242/jcs.264577#supplementary-data

Table S4. Mammalian and bacterial expression plasmids

Name	Insert	Promoter	Source
pCE32	2xFKBP-msGFP2-ULK1	CMV10	Eickhorst et al., 2024
pCE38	mt-mKeima-P2A-FRB-FIS1	CMV10	Addgene # 135295
pCE113	2xFKBP-msGFP2	CMV10	Eickhorst et al., 2024
pCE23	Flp recombinase	CMV10	Invitrogen, # 35–3018
pRB7	2xFKBP-myc-APEX2-ULK1	CMV10	Licheva et al., 2025
pRB30	2xFKBP-myc-APEX2	CMV10	Licheva et al., 2025
pRB44	2xFKBP-myc-APEX2-ULK1-K46I	CMV10	This study
pRB47	MTFR1L-V5	CMV10	Addgene plasmid # 69822
pRB57	MTFR1L-mScarlet	CMV10	This study
pCE70	2xFKBP-msGFP2-ULK1 for Flp-In T-REx	CMV10	Licheva et al., 2025
pCE167	2xFKBP-msGFP2-ULK1 K46I for Flp-In T-REx	CMV10	This study
pCE168	FRB-FIS1 (93–152)	CMV10	Licheva et al., 2025
pCE271	Parkin, stable integration	CMV10	This study
pRB159	FIS1$^{93\text{-}152}$-MTFR1L	CMV10	This study
pRB161	FIS1$^{93\text{-}152}$-mScarlet	CMV10	This study

Journal of Cell Science • Supplementary information

Table S5. Mammalian cell lines

Name	Cell line	Gene KO	Stably inserted plasmids	Source
cCE175	U2OS Flp-In T-REx mt-mKeima, FRB-FIS1 (93–152)	-	pCE38	Eickhorst et al., 2024
cPS66	U2OS Flp-In T-REx mt-mKeima, FRB-FIS1 (93–152), Parkin	-	pCE38, pCE271	Eickhorst et al., 2024
cCE377	U2OS Flp-In T-REx mt-mKeima, FRB-FIS1 (93–152), 2xFKBP-GFP-ULK1	-	pCE38, pCE70	Licheva et al., 2025
cCE308	U2OS Flp-In T-REx, FRB-FIS1 (93–152)	-	pCE168	Licheva et al., 2025
cCE403	U2OS Flp-In T-REx mt-mKeima, FRB-FIS1 (93–152), 2xFKBP-GFP-ULK1-K46I	-	pCE38, pCE167	This study
cRB12	HEK293 Flp-In T-REx, mt-mKeima, FRB-FIS1 (93–152), 2xFKBP-myc-APEX2	-	pCE38, pRB30	Licheva et al., 2025
cRB7	HEK293 Flp-In T-REx, mt-mKeima, FRB-FIS1 (93–152), 2xFKBP-myc-APEX2-ULK1	-	pCE38, pRB7	Licheva et al., 2025
cRB20	HEK293 Flp-In T-REx, mt-mKeima, FRB-FIS1 (93–152), 2xFKBP-myc-APEX2-ULK1-K46I	-	pCE38, pRB44	This study
cCE27	U2OS Flp-In T-REx	-	-	Jakob Nilsson, University of Copenhagen, Denmark

Table S6. E.coli expression plasmids

Name	Characteristics	Promoter	Source
pFK32	GST	pGEX5x2	GE Healthcare
SMc295	GST-ATG19 (365-415)	pGEX5x2	Sascha Martens lab
pRB65	GST-MTFR1L (1-20)	pGEX5x2	This study
pRB66	GST-MTFR1L (170-189)	pGEX5x2	This study
pRB67	GST-MTFR1L (1-20; T7A)	pGEX5x2	This study
pRB68	GST-MTFR1L (170-189; S177A)	pGEX5x2	This study
pRB94	GST-MTFR1L (159-171)	pGEX5x2	This study
pRB95	GST-MTFR1L (159-171; S165A)	pGEX5x2	This study

Journal of Cell Science • Supplementary information
