# Supplementary material for: A ULK1–MTFR1L feedback loop links mitochondrial fission, mitophagy and apoptosis
Source: J Cell Sci. 2026 May 11;139(9):jcs264577. doi: 10.1242/jcs.264577 (PMC13245916; doi:10.1242/jcs.264577)
Supplement: Supplementary file 1 [file joces-139-264577_review_history.pdf]

A ULK1-MTFR1L feedback loop links mitochondrial fission, mitophagy and apoptosis

Riccardo Babic, Leon Lucya, Christoph Reiter, Franziska Kriegenburg, Ramón Castellanos-Martínez, David M. Hollenstein, Florian Becker, Carola Hunte and Claudine Kraft
DOI: 10.1242/jcs.264577

Editor: Tamotsu Yoshimori

Review timeline

Original submission:	7 November 2025
Editorial decision:	15 December 2025
First revision received:	18 March 2026
Editorial decision:	7 April 2026
Second revision received:	7 April 2026
Accepted:	8 April 2026

Original submission

First decision letter

MS ID#: jcs.264577

MS TITLE: A ULK1-MTFR1L feedback loop links mitochondrial fission, mitophagy and apoptosis

AUTHORS: Riccardo Babic; Leon Lucya; Christoph Reiter; Franziska Kriegenburg; David M Hollenstein; Florian Becker; Carola Hunte; Claudine Kraft

ARTICLE TYPE: Research Article

Dear Dr Kraft,

We have now reached a decision on the above manuscript.

As you will see, the reviewers raise a number of substantial criticisms that prevent me from accepting the paper at this stage. They suggest, however, that a revised version might prove acceptable, if you can address their concerns. If you think that you can deal satisfactorily with the criticisms on revision, I would be pleased to see a revised manuscript. We would then return it to the reviewers.

Reviewer 1

Advance summary and potential significance to field

Mitophagy preserves mitochondrial quality, yet how mitochondrial fission is coordinated with autophagy initiation remains unclear. Using a synthetic FKBP-FRB system to tether ULK1 kinase to mitochondria independently of damage, the authors found that MTFR1L was enriched at ULK1 foci together with additional fission factors. Moreover, gain-of-function assays show that mitochondrial targeting of MTFR1L increases mitochondrial fragmentation, whereas MTFR1L depletion decreases respiratory capacity, elevates apoptosis, and impairs mitophagy flux. MTFR1L co-localizes with the ULK1 complex. Upon mitophagy induction, MTFR1L is phosphorylated in a ULK1 kinase-dependent

© 2026. Published by The Company of Biologists under the terms of the Creative Commons Attribution License (https://creativecommons.org/licenses/by/4.0/).

manner, and reciprocally modulates ULK1 activity, establishing a feedback loop. These findings position MTFR1L as a critical link between mitochondrial fission and the autophagy machinery. Conclusively, the mitochondrial outer membrane protein MTFR1L seems a key component of mitophagy initiation hubs.

Throughout the manuscript, a major weakness of this manuscript is that the precise molecular function of MTFR1L remains somewhat vague, and the mechanistic explanation is lacking. Nevertheless, the data are generally clean, and the manuscript is well written without unnecessary descriptions. Therefore, I would like to support publication after revision, but the following weaknesses should be addressed. Points (3) and (5) require additional experiments.

(1) Figure 1E is presented as evidence that MTFR1L promotes mitochondrial fission. However, the observed difference compared with the control—although statistically significant—is exceedingly small. Moreover, it remains unclear whether the conclusions of the prior study by Tilokani et al. (2022) are indeed correct. It would be valuable for the authors (and for us) to examine whether the previous findings by Tilokani et al. can be independently validated. Given this, is it appropriate for the authors to conclude that MTFR1L promotes mitochondrial fission based on such a minimal difference?

(2) The data in Fig. 2D clearly demonstrate that loss of MTFR1L function induces apoptotic cell death and elevated cleavage of PARP and caspase-3. Could the authors provide a deeper discussion of this finding? If there are constraints such as limitation of the manuscript length, briefly explain in the rebuttal letter.

(3) To understand MTFR1L's function, experiments using MTFR1L-deficient cells are essential. The authors provide reasons for not using KO cells and depend solely on knockdown. Although I do not dismiss this approach, to exclude off-target effects, rescue experiments are absolutely necessary. Although the authors performed a rescue experiment in Fig. 3C, they should also perform the same rescue experiments for Fig. 2D and Fig. 5C.

(4) The mt-mKeima-based mitophagy assay shows that inhibition of MTFR1L impaired mitophagy (Fig. 3), and this is a key result of the manuscript. To evaluate the validity of this conclusion, the rescue experiment shown in Fig. 3C is particularly important. Nevertheless, the manuscript does not explain why the reintroduced MTFR1L is resistant to the siRNA. Was a silent mutation introduced into the siRNA target sequence, or is siRNA #1 directed against a non-coding region? The authors should clarify in the main text how the reintroduced MTFR1L was made siRNA-resistant.

(5) The data demonstrating MTFR1L is phosphorylated by ULK1 (Fig. 5) are insufficiently validated. First, the authors should explain why a full-length MTFR1L fused to GST was not used as a substrate in Fig. 5B.
Next, since the authors identified putative phosphorylation sites, the following mutational experiments should be performed:
The authors should generate three types siRNA-resistant MTFR1L mutants—MTFR1L(T7A, S177A), MTFR1L(T7D, S177D), and MTFR1L(T7E, S177E). Then these mutants should be introduced into MTFR1L-siRNA cells to examine how they affect complementation activity (Fig. 3C) as well as ULK1-dependent phosphorylation of MTFR1L in cells (Fig. 4).

Ideally, the following results would be expected:
(i) WT MTFR1L is phosphorylated and complements the phenotype observed upon MTFR1L knockdown.
(ii) The phosphorylation-deficient MTFR1L(T7A, S177A) mutant is not phosphorylated and fails to complement the knockdown phenotype.
(iii) The phosphomimetic mutants MTFR1L(T7D, S177D) and (T7E, S177E) is not phosphorylated but is able to complement the knockdown phenotype.
Of course, I fully recognize that not all phosphorylated substrates yield results that perfectly match these expectations; conversely, in some cases—such as the case in PINK1 autophosphorylation—phosphorylation deficient and phosphomimetic mutations showed the anticipated outcomes. Even if these experiments do not yield the ideal results, I would not consider this grounds for rejection. However, given that the authors claim to have identified the phosphorylation sites of MTFR1L, such experiments should be performed.

© 2026. Published by The Company of Biologists under the terms of the Creative Commons Attribution License (https://creativecommons.org/licenses/by/4.0/).

Reviewer 2

Advance summary and potential significance to field

In this manuscript, the authors focus on MTFR1L, a mitochondrial fission-related factor, identified through an analysis of proteins in proximity to ULK1 during mitophagy. They report that MTFR1L and ULK1 cooperate to form mitophagy initiation hubs. In addition, they find that MTFR1L knockdown (siMTFR1L) reduces mitochondrial activity, increases susceptibility to apoptosis, and suppresses mitophagy. Furthermore, because ULK1 phosphorylates MTFR1L and MTFR1L in turn affects ULK1 activity, the authors argue that crosstalk between MTFR1L and ULK1 impacts mitochondrial fission, mitophagy, and apoptosis.
Each individual experiment appears to be carefully performed, and the overall claims are not inconsistent with the presented data. However, the conclusions are not yet convincingly demonstrated. In particular, the following points require careful attention.

Specific comments

Figure 1F: When FKBP-GFP-ULK1 is targeted to mitochondria by rapalog, what is the resulting mitochondrial morphology? If MTFR1L-mScarlet can be considered a marker of mitochondrial morphology, mitochondria appear highly punctate. Should this be interpreted as enhanced mitochondrial fission?
In addition, are the puncta where FKBP-GFP-ULK1 and MTFR1L-mScarlet colocalize mitochondria already engulfed by autophagosomes, or do they represent a pre-engulfment state (prior to or during phagophore formation)?
Moreover, in rapalog treated cells expressing FKBP-GFP (without ULK1), both FKBP-GFP and MTFR1L-mScarlet should localize to mitochondria. FKBP-GFP appears enriched near the nucleus. It is not clear why this localization pattern is observed, nor is it entirely clear whether these signals correspond to mitochondria. However, for mitochondria located farther from the nucleus, FKBP-GFP seems to fully colocalize with MTFR1L-mScarlet. Given this, could one interpret that FKBP-GFP colocalizes with MTFR1L-mScarlet independently of ULK1?

MTFR1L phosphorylation is clear in cells expressing FKBP-GFP-ULK1 (Figure 4A), but can MTFR1L phosphorylation also be detected in cells expressing only endogenous ULK1? If so, can this phosphorylation be suppressed by ULK1 knockout or knockdown?

If ULK1 phosphorylates MTFR1L and MTFR1L affects ULK1 activity, the interaction between MTFR1L and the ULK1 complex might be relatively robust. Can this interaction be demonstrated by co-immunoprecipitation?

The authors show that MTFR1L-FIS1 expression promotes mitochondrial fission, but how does siMTFR1L affect mitochondrial morphology?

In this study, mitophagy is quantified by flow cytometry of cells expressing mt-Keima, which is a widely used approach. However, when mitochondrial membrane potential and/or overall cellular function is compromised, mt-Keima may fail to localize properly to mitochondria. Therefore, as in the original Katayama et al. paper, it would be important to verify mitochondrial morphology in the neutral (non-acidic) mt-Keima channel by fluorescence microscopy, and then quantify punctate acidic lysosomal signals that contain mitochondria.

First revision

Author response to reviewers' comments

© 2026. Published by The Company of Biologists under the terms of the Creative Commons Attribution License (https://creativecommons.org/licenses/by/4.0/).

Point-by-point response to reviewer comments

We thank the reviewers for their thoughtful and constructive feedback, which has helped us improve the manuscript. We have addressed all comments point by point below and revised the manuscript accordingly. We hope that the reviewers will find the work suitable for publication.

Reviewer 1:

Comments from the Reviewers:

> *Reviewer 1: Mitophagy preserves mitochondrial quality, yet how mitochondrial fission is coordinated with autophagy initiation remains unclear. Using a synthetic FKBP-FRB system to tether ULK1 kinase to mitochondria independently of damage, the authors found that MTFR1L was enriched at ULK1 foci together with additional fission factors. Moreover, gain-of-function assays show that mitochondrial targeting of MTFR1L increases mitochondrial fragmentation, whereas MTFR1L depletion decreases respiratory capacity, elevates apoptosis, and impairs mitophagy flux. MTFR1L co-localizes with the ULK1 complex. Upon mitophagy induction, MTFR1L is phosphorylated in a ULK1 kinase-dependent manner, and reciprocally modulates ULK1 activity, establishing a feedback loop. These findings position MTFR1L as a critical link between mitochondrial fission and the autophagy machinery. Conclusively, the mitochondrial outer membrane protein MTFR1L seems a key component of mitophagy initiation hubs.*
>
> *Throughout the manuscript, a major weakness of this manuscript is that the precise molecular function of MTFR1L remains somewhat vague, and the mechanistic explanation is lacking. Nevertheless, the data are generally clean, and the manuscript is well written without unnecessary descriptions. Therefore, I would like to support publication after revision, but the following weaknesses should be addressed.*
> *Points (3) and (5) require additional experiments.*
>
> *(1) Figure 1E is presented as evidence that MTFR1L promotes mitochondrial fission. However, the observed difference compared with the control—although statistically significant—is exceedingly small. Moreover, it remains unclear whether the conclusions of the prior study by Tilokani et al. (2022) are indeed correct. It would be valuable for the authors (and for us) to examine whether the previous findings by Tilokani et al. can be independently validated. Given this, is it appropriate for the authors to conclude that MTFR1L promotes mitochondrial fission based on such a minimal difference?*

To strengthen our conclusion, we have included an additional morphological metric beyond average edge length.
Specifically, we have added quantification of the ratio of free mitochondrial ends to total mitochondrial length, which provides a measure of the degree of fragmentation (i.e., the relative abundance of smaller, untethered mitochondrial pieces). This metric also shows a statistically significant increase in cells expressing FIS193-152
-
MTFR1L compared to the mScarlet control, further supporting a shift toward fragmentation (Figure 1E). We have also added further representative images in the supplement (Figure S1A).
We have also softened our statement, replacing “promotes mitochondrial fission” with “shifts mitochondrial morphology toward fragmentation.” We note that the published Tilokani et al. (2022) study, which used the same siRNA, showed hyperfusion upon MTFR1L depletion, independently supporting the idea that MTFR1L influences the fission/fusion balance. Our gain-of-function data are consistent with this model.
Furthermore, a study published during the revision of this manuscript demonstrates that MTFR1L

© 2026. Published by The Company of Biologists under the terms of the Creative Commons Attribution License (https://creativecommons.org/licenses/by/4.0/).

binds phospho-S65-ubiquitin and is required for efficient PINK1/Parkin-dependent mitophagy in the heart in vivo, providing independent mechanistic evidence that MTFR1L plays an active role in mitophagy initiation beyond its function in mitochondrial morphology (Shi et al., PNAS 2026). Together, these independent lines of evidence support the biological significance of MTFR1L in mitochondrial quality control and strengthen the conclusion that the effects we observe in our gain-of-function and loss-of-function experiments reflect MTFR1L biology.

(2) The data in Fig. 2D clearly demonstrate that loss of MTFR1L function induces apoptotic cell death and elevated cleavage of PARP and caspase-3. Could the authors provide a deeper discussion of this finding? If there are constraints such as limitation of the manuscript length, briefly explain in the rebuttal letter.

As suggested by the reviewer, we added a deeper discussion to these findings.

(3) To understand MTFR1L's function, experiments using MTFR1L-deficient cells are essential. The authors provide reasons for not using KO cells and depend solely on knockdown. Although I do not dismiss this approach, to exclude off-target effects, rescue experiments are absolutely necessary. Although the authors performed a rescue experiment in Fig. 3C, they should also perform the same rescue experiments for Fig. 2D and Fig. 5C.

To substantiate that the observed phenotypes are not occurring by off-target effects, we have performed further rescue experiments. As expected, re-expression of MTFR1L in siMTFR1L-treated cells partly rescues the apoptotic phenotype observed in Fig. 2D, as cells re-expressing MTFR1L show a reduction in cleaved caspase-3 and cleaved PARP compared to cells treated with siMTFR1L alone. These data have been added as new Figure 2E and support that the observed phenotype results from MTFR1L depletion rather than off-target effects. During our experiments, we have however observed that reexpression of MTFR1L can be challenging, as too much and too little of the protein affect cell function (see comment (4) below). Therefore, a partial rescue rather than full rescue is expected. Also, the use of 3 different siRNA targeting MTFR1L showed a similar decrease in the mitophagy flux, further supporting that the phenotype is due to MTFR1L depletion rather than off-target effects (Figure 3D).

(4) The mt-mKeima-based mitophagy assay shows that inhibition of MTFR1L impaired mitophagy (Fig. 3), and this is a key result of the manuscript. To evaluate the validity of this conclusion, the rescue experiment shown in Fig. 3C is particularly important. Nevertheless, the manuscript does not explain why the reintroduced MTFR1L is resistant to the siRNA. Was a silent mutation introduced into the siRNA target sequence, or is siRNA #1 directed against a non-coding region? The authors should clarify in the main text how the reintroduced MTFR1L was made siRNA-resistant.

We apologize for the confusion, we would like to clarify that in the rescue experiments shown in the manuscript, we used a non-siRNA-resistant MTFR1L construct. Because siRNA #1 does not achieve complete knockdown when exogenous MTFR1L is simultaneously overexpressed, sufficient protein levels were maintained despite siRNA treatment. While this introduced some experimental variability, it was the most reliable approach in our hands and consistently produced detectable MTFR1L expression alongside effective endogenous knockdown.

Attempts to use a fully siRNA-resistant version of MTFR1L (in which silent mutations were introduced into the siRNA #1 target sequence to prevent siRNA binding without altering the protein sequence) presented unexpected challenges. When expressed at fully protected levels, MTFR1L protein accumulated at 4-5 fold above endogenous levels and failed to rescue the MTFR1L-dependent phenotypes. We attribute this failure to the overexpression levels, which led to aggregate formation visible by fluorescence microscopy, consistent with the known instability of recombinant

© 2026. Published by The Company of Biologists under the terms of the Creative Commons Attribution License (https://creativecommons.org/licenses/by/4.0/).

MTFR1L. This suggests that MTFR1L function is highly sensitive to protein dosage and requires expression within a narrow physiological range. We therefore used the non-resistant construct for all rescue experiments, which, by virtue of partial siRNA-mediated suppression of the exogenous construct, maintained MTFR1L expression closer to physiological levels and consistently produced detectable rescue.

(5) The data demonstrating MTFR1L is phosphorylated by ULK1 (Fig. 5) are insufficiently validated. First, the authors should explain why a full-length MTFR1L fused to GST was not used as a substrate in Fig. 5B. Next, since the authors identified putative phosphorylation sites, the following mutational experiments should be performed:
The authors should generate three types siRNA-resistant MTFR1L mutants—MTFR1L(T7A, S177A), MTFR1L(T7D, S177D), and MTFR1L(T7E, S177E). Then these mutants should be introduced into MTFR1L-siRNA cells to examine how they affect complementation activity (Fig. 3C) as well as ULK1- dependent phosphorylation of MTFR1L in cells (Fig. 4). Ideally, the following results would be expected:
WT MTFR1L is phosphorylated and complements the phenotype observed upon MTFR1L knockdown.
The phosphorylation-deficient MTFR1L(T7A, S177A) mutant is not phosphorylated and fails to complement the knockdown phenotype. (iii) The phosphomimetic mutants MTFR1L(T7D, S177D) and (T7E, S177E) is not phosphorylated but is able to complement the knockdown phenotype. Of course, I fully recognize that not all phosphorylated substrates yield results that perfectly match these expectations; conversely, in some cases—such as the case in PINK1 autophosphorylation—phosphorylation deficient and phosphomimetic mutations showed the anticipated outcomes. Even if these experiments do not yield the ideal results, I would not consider this grounds for rejection. However, given that the authors claim to have identified the phosphorylation sites of MTFR1L, such experiments should be performed.

We initially attempted to use full-length GST-MTFR1L in the in vitro kinase assay. However, two significant obstacles prevented this approach:

First, the molecular weight of full-length GST-MTFR1L (~65 kDa) overlaps with the region of the autoradiograph where autophosphorylation of the recombinant ULK1 kinase complex is most prominent. This makes it difficult to reliably attribute any radiolabeled signal in this region to MTFR1L phosphorylation rather than ULK1 autophosphorylation. Attempts to recombinantly express MTFR1L and to remove the solubility tag (including GST, MBP and Strep-NT*) result in the immediate degradation of the MTFR1L protein.
Consistent with this intrinsic instability, a recent study identified MTFR1L as a target for oxidative cleavage, which generates a C-terminal amide 'degron' recognized by the SCF–FBXO31 ubiquitin ligase pathway (Muhar et al., Nature 2025). This suggests that MTFR1L may be particularly susceptible to backbone fragmentation and subsequent rapid turnover when expressed in non-native conditions.
Second, and surprisingly, full-length GST-MTFR1L in its isolated form was found to strongly inhibit the in vitro kinase reaction itself, as evidenced by markedly diminished ULK1 autophosphorylation in its presence. This unexpected inhibitory effect precluded use of the full-length substrate entirely.

© 2026. Published by The Company of Biologists under the terms of the Creative Commons Attribution License (https://creativecommons.org/licenses/by/4.0/).

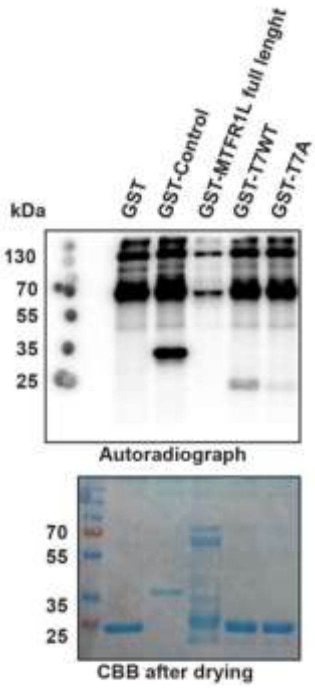

We therefore used short GST-tagged peptide fragments encompassing the individual candidate phosphorylation sites, an approach that has been successfully employed in previous ULK1 substrate studies. The specificity of the assay at the individual candidate phosphorylation sites (T7 and S177) was validated by the inability of ULK1 to phosphorylate the neighboring S165 residue, which does not match the ULK1 consensus motif.

As requested, we performed rescue experiments using both the phosphorylation-deficient double alanine mutant MTFR1L (T7A, S177A) and the phosphomimetic double aspartate mutant MTFR1L (T7D, S177D). As shown in new Figure 5C, both the AA and DD mutants rescue the mitophagy flux defect to levels comparable to wild-type MTFR1L re-expression. This indicates that neither mutant has a dominant-negative or constitutively activating effect on mitophagy in the set-up used after ULK1 tethering. Given that MTFR1L is phosphorylated upon ULK1 tethering and that tethering induces mitophagy, one might have expected the phosphomimetic mutant to show increased mitophagy flux. This suggests that the phosphorylation at T7 and S177 may not be strictly required for the scaffolding function of MTFR1L that supports mitophagy, but may instead fine-tune the ULK1-MTFR1L interaction. It should also be noted that MTFR1L contains 52 serine and threonine residues in total. A comprehensive phosphoproteomic scan across the full protein, ideally under conditions of ULK1 activation, will be necessary to fully map the phosphorylation landscape of MTFR1L and determine which additional sites are functionally critical. This is however beyond the scope of this study. We nevertheless included the new data and added this suggestion in the discussion, and adapted the final model accordingly to reflect the possibility of regulation by additional kinases beyond ULK1, consistent with the previously proposed role of AMPK.

Reviewer 2:

Reviewer 2: In this manuscript, the authors focus on MTFR1L, a mitochondrial fission-related factor, identified through an analysis of proteins in proximity to ULK1 during mitophagy. They report that MTFR1L and ULK1 cooperate to form mitophagy initiation hubs. In addition, they find that MTFR1L knockdown (siMTFR1L) reduces mitochondrial activity, increases susceptibility to apoptosis, and suppresses mitophagy. Furthermore, because ULK1 phosphorylates MTFR1L and MTFR1L in turn affects ULK1 activity, the authors argue that crosstalk between MTFR1L and ULK1 impacts

© 2026. Published by The Company of Biologists under the terms of the Creative Commons Attribution License (https://creativecommons.org/licenses/by/4.0/).

mitochondrial fission, mitophagy, and apoptosis.
Each individual experiment appears to be carefully performed, and the overall claims are not inconsistent with the presented data. However, the conclusions are not yet convincingly demonstrated. In particular, the following points require careful attention.

Specific comments

Figure 1F: When FKBP-GFP-ULK1 is targeted to mitochondria by rapalog, what is the resulting mitochondrial morphology? If MTFR1L-mScarlet can be considered a marker of mitochondrial morphology, mitochondria appear highly punctate. Should this be interpreted as enhanced mitochondrial fission?
In addition, are the puncta where FKBP-GFP-ULK1 and MTFR1L-mScarlet colocalize mitochondria already engulfed by autophagosomes, or do they represent a pre-engulfment state (prior to or during phagophore formation)?
Moreover, in rapalog treated cells expressing FKBP-GFP (without ULK1), both FKBP-GFP and MTFR1L- mScarlet should localize to mitochondria. FKBP-GFP appears enriched near the nucleus. It is not clear why this localization pattern is observed, nor is it entirely clear whether these signals correspond to mitochondria. However, for mitochondria located farther from the nucleus, FKBP-GFP seems to fully colocalize with MTFR1L-mScarlet. Given this, could one interpret that FKBP-GFP colocalizes with MTFR1L-mScarlet independently of ULK1?

To address the mitochondria morphology upon tethering, we have added a new panel (Figure 1G) showing the mitochondrial network stained with MitoTrackerDeepRed in cells expressing FKBP-GFP-ULK1 following rapalog treatment. This allows direct visualization of mitochondrial morphology independently of the MTFR1L-mScarlet signal. The data show that upon short ULK1 tethering there are no major mitochondrial network changes. We also refer the reviewer to Figure 3E of Eickhorst et al. JMB (2024), which analyses mitochondrial morphology in this synthetic tethering system in more detail. Representative images comparing cells with and without rapalog treatment, showing MitoTrackerDeepRed signal, are provided below.

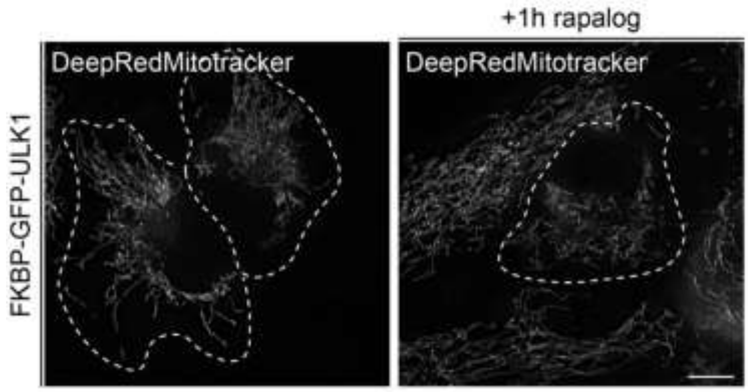

We would also like to clarify that we did not intend to use MTFR1L-mScarlet as a morphological readout of the mitochondrial network in Figure 1F. MTFR1L-mScarlet was used in that experiment solely to assess its localization relative to ULK1 foci, not as a proxy for mitochondrial morphology. As this seems to have been misleading, we have now added Figure 1G showing MitoTrackerDeepRed staining alongside the existing channels, which provides an unambiguous and independent readout of the mitochondrial network.

Regarding whether the puncta where FKBP-GFP-ULK1 and MTFR1L-mScarlet colocalize represent pre- or post- engulfment mitochondria: since rapalog treatment continuously triggers mitophagy initiation without any

© 2026. Published by The Company of Biologists under the terms of the Creative Commons Attribution License (https://creativecommons.org/licenses/by/4.0/).

pharmacological block, the population of ULK1 puncta observed at any given timepoint is expected to represent multiple stages of the mitophagy pathway simultaneously, ranging from early initiation hubs prior to phagophore formation, to mitochondria already in the process of being engulfed. This is inherent to the proximity proteomics approach used to identify MTFR1L (Figure 1D), in which biotinylation captures the local environment of ULK1 across all stages of mitophagy without stage-specific arrest. Consistent with this, MTFR1L was enriched in the vicinity of the kinase-dead ULK1-K46I, which stalls mitophagy at an early pre-engulfment stage, supporting the interpretation that at least a subset of the observed colocalization reflects early initiation prior to autophagosome engulfment.

Upon re-examination, the apparent perinuclear enrichment of FKBP-GFP in the original representative image was specific to the cell chosen as an example. We have replaced this image with a more representative example. We would also like to clarify an important aspect of the experimental design that may have caused confusion: in this system, FKBP-GFP is itself tethered to the outer mitochondrial membrane via rapalog-induced dimerization with FRB-FIS193-152. Therefore, upon rapalog addition, FKBP-GFP (without ULK1) localizes to mitochondria and distributes broadly across the entire mitochondrial network, as does MTFR1L-mScarlet. The observed colocalization between FKBP-GFP and MTFR1L-mScarlet in this control condition thus simply reflects that both proteins are present on mitochondria, distributed across the network, and is not indicative of a specific interaction. The key distinction is therefore not between colocalization and non-colocalization in the control, but between a diffuse, network-wide colocalization (FKBP-GFP control) and the formation of discrete, punctate foci (FKBP-GFP- ULK1 with rapalog). Upon ULK1 tethering, FKBP-GFP-ULK1 concentrates into specific mitophagy initiation hubs, and MTFR1L-mScarlet is also selectively enriched at these hubs, a focal colocalization that is quantitatively distinct from the uniform network colocalization seen in the control. This distinction is quantified in the quantification graph (Figure 1F), which scores the percentage of cells with discrete FKBP-GFP-ULK1 foci colocalizing with MTFR1L- mScarlet, a metric that specifically reports the hub-associated colocalization and not the general mitochondrial overlap.

MTFR1L phosphorylation is clear in cells expressing FKBP-GFP-ULK1 (Figure 4A), but can MTFR1L phosphorylation also be detected in cells expressing only endogenous ULK1? If so, can this phosphorylation be suppressed by ULK1 knockout or knockdown?

To address this, we performed the experiment using Antimycin A and Oligomycin (AO) treatment to induce mitophagy via endogenous ULK1, and analyzed MTFR1L mobility by both standard SDS-PAGE and Phos-tag gels. In addition, to directly test whether any AO-induced MTFR1L phosphorylation is ULK1-dependent, we included a siULK1 condition alongside the siControl. As shown below, we did not observe a comparable mobility shift of MTFR1L under AO conditions in either siControl or siULK1 cells, compared to the tethering experiment, which served as a positive control. Consistent with efficient knockdown, ULK1 protein levels were markedly reduced in the siULK1 condition as confirmed by standard SDS-PAGE.
We interpret this result as reflecting the mechanistic difference between the two induction systems: in the synthetic tethering model, a high local concentration of ULK1 is delivered directly to mitochondria, potentially resulting in more efficient phosphorylation of MTFR1L than is achievable by endogenous ULK1 during damage-induced mitophagy. This is consistent with the idea that ULK1-MTFR1L phosphorylation is a transient event under physiological conditions, making it technically challenging to detect without signal amplification or stalling the process at initiation.

© 2026. Published by The Company of Biologists under the terms of the Creative Commons Attribution License (https://creativecommons.org/licenses/by/4.0/).

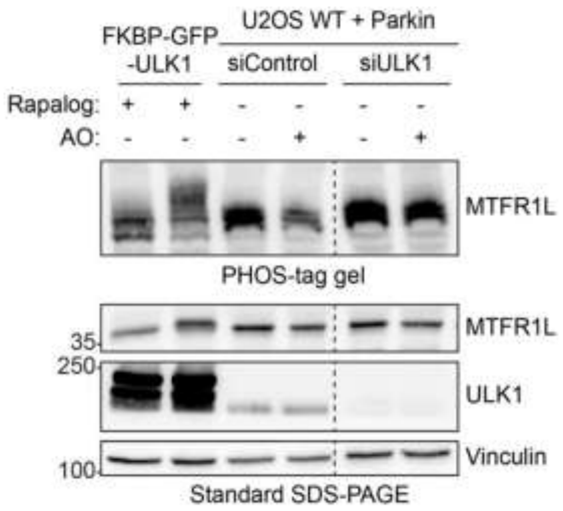

If ULK1 phosphorylates MTFR1L and MTFR1L affects ULK1 activity, the interaction between MTFR1L and the ULK1 complex might be relatively robust. Can this interaction be demonstrated by co- immunoprecipitation?

We have performed the suggested co-immunoprecipitation experiments using GFP-Trap pulldown of FKBP-GFP- ULK1. Under these conditions, we could co-immunoprecipitate all ULK1 complex members (ATG13, FIP200, ATG101), but we did not detect MTFR1L in the pulldown (see figure below). Negative controls (e.g., vinculin) were not enriched, confirming the specificity of the pulldown for the ULK1 complex. We also performed the reciprocal experiment using an anti-MTFR1L antibody for pulldown, with similar results.

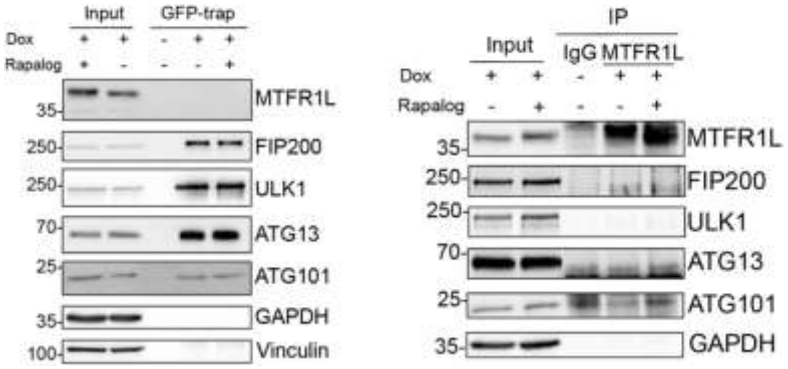

Taken together, these co-IP data suggest that the MTFR1L-ULK1 interaction is transient or of low affinity, which is not unusual for kinase-substrate relationships or for interactions that occur specifically at mitochondrial membranes and may be disrupted by detergent-based lysis. We believe that additional experiments would be required to demonstrate this interaction convincingly by co-IP. However, proximity analysis is more suitable for transient or low-affinity interactions. The proximity proteomics data (Figure 1D), which show robust enrichment of MTFR1L in the vicinity of ULK1-K46I stalled at initiation hubs, provide stronger and more direct evidence for their spatial association in situ, and are also well suited to capture transient interactions.

The authors show that MTFR1L-FIS1 expression promotes mitochondrial fission, but how does siMTFR1L affect mitochondrial morphology?

To address this point, we have added MitoTrackerDeepRed staining to Figure 3F, allowing direct visualization of the mitochondrial network in siControl and siMTFR1L cells. In addition, we performed MitoGraph analysis of MitoTrackerDeepRed-stained siControl versus siMTFR1L cells and include the results in the figure below. However,

© 2026. Published by The Company of Biologists under the terms of the Creative Commons Attribution License (https://creativecommons.org/licenses/by/4.0/).

we note that the quantitative analysis is technically complicated by the phenotypic consequences of MTFR1L depletion itself. After 72h of siRNA treatment, siMTFR1L cells show reduced viability and lower metabolic activity (Figure 2B-C), resulting in reduced cell density and larger individual cells with more mitochondria compared to siControl. To account for this, we restricted the comparison to cells of comparable size.
Under these conditions, average mitochondrial edge length as well as other metrics were essentially identical between siControl and siMTFR1L cells. Only the free ends to total network length ratio showed a tendency toward a decrease in siMTFR1L cells, consistent with a more interconnected network. We therefore do not feel confident making a definitive statement about hyperfusion in the manuscript, as a robust conclusion would require deeper and more comparable analysis controlling for the other effects of reduced cell fitness. Consistently, visual inspection of the representative microscopy images (Figure 3F) does not reveal a striking morphological difference between siControl and siMTFR1L cells, in contrast to the clear and obvious fragmentation visible in the gain-of- function experiment (Fig. 1E). We would like to point out that Tilokani et al. (2022) reported a hyperfusion phenotype using photoactivation of mitochondrially-targeted PA-GFP (mito-PA-GFP), a method that measures the rate of fluorescence spread through the mitochondrial network. The slight tendency we observe in the free ends metric is consistent with their findings, and the discrepancy in effect magnitude may be explained by the methodological difference.

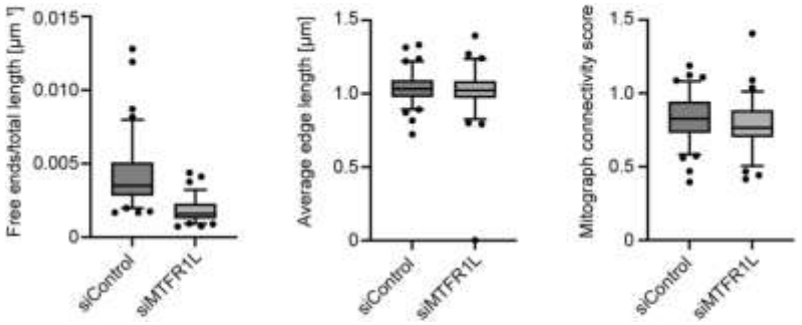

In this study, mitophagy is quantified by flow cytometry of cells expressing mt-Keima, which is a widely used approach. However, when mitochondrial membrane potential and/or overall cellular function is compromised, mt-Keima may fail to localize properly to mitochondria. Therefore, as in the original Katayama et al. paper, it would be important to verify mitochondrial morphology in the neutral (non-acidic) mt-Keima channel by fluorescence microscopy, and then quantify punctate acidic lysosomal signals that contain mitochondria.

We have performed the requested microscopy validation and added new Figure 3E to the manuscript. In this figure, we show that in cells under nutrient-rich conditions:
(i) The neutral-pH mt-mKeima signal colocalizes with MitoTrackerDeepRed, confirming that mt-mKeima correctly localizes to mitochondria under basal conditions in both siControl and siMTFR1L cells.
(ii) Discrete acidic puncta are detectable in the 561 nm excitation channel, and these puncta always colocalize with LysoTracker in both siControl and siMTFR1L, confirming their lysosomal identity.
These data confirm that the mt-mKeima assay is reporting mitophagy flux and is not confounded by mislocalization of the mKeima protein.

<u>Second decision letter</u>

© 2026. Published by The Company of Biologists under the terms of the Creative Commons Attribution License (https://creativecommons.org/licenses/by/4.0/).

MS ID#: jcs.264577R1

MS TITLE: A ULK1-MTFR1L feedback loop links mitochondrial fission, mitophagy and apoptosis

AUTHORS: Riccardo Babic; Leon Lucya; Christoph Reiter; Franziska Kriegenburg; Ramón Castellanos-Martínez; David M Hollenstein; Florian Becker; Carola Hunte; Claudine Kraft

ARTICLE TYPE: Research Article

Dear Dr Kraft,

We have now reached a decision on the above manuscript.

As you will see, the reviewers gave favourable reports; however, since Reviewer #1 has requested minor revisions, please address their comments.

Second revision

Author response to reviewers' comments

Point-by-point reply

Minor points
(1) The following sentence appears to contain an error:
"We examined whether MTFR1L influences the mitophagy flux using mitochondrial-targeted mKeima (mt-mKeima), a fluorophore with dual excitation properties that distinguishes between neutral and acidic pH environments 15.03.26 19:23:00."
What does the final number "15.03.26 19:23:00" refer to? It should be removed or clarified.

This seems to have been a field code error during conversion. We have pasted that region as plain text, which resolved the issue.

(2) There is an inconsistency regarding figure citation:
Although Fig. 3E is referred to in the rebuttal letter, its description is missing in the main text (it may have been mistakenly labeled as Fig. 3F in the revision).

We thank the reviewer for noting this, indeed, the first mentioned 3F should be 3E.

Third decision letter

MS ID#: jcs.264577R2

MS Title: A ULK1-MTFR1L feedback loop links mitochondrial fission, mitophagy and apoptosis

Authors: Riccardo Babic; Leon Lucya; Christoph Reiter; Franziska Kriegenburg; Ramón Castellanos-Martínez; David M Hollenstein; Florian Becker; Carola Hunte; Claudine Kraft

Article Type: Research Article

Dear Dr Kraft,

I am happy to tell you that your manuscript has been accepted for publication in Journal of Cell Science, pending standard publication integrity checks.

© 2026. Published by The Company of Biologists under the terms of the Creative Commons Attribution License (https://creativecommons.org/licenses/by/4.0/).

© 2026. Published by The Company of Biologists under the terms of the Creative Commons Attribution License (https://creativecommons.org/licenses/by/4.0/).
